# Multiplexed embryo profiling links cellular state to zygotic genome activation in single cells

**DOI:** 10.1038/s41467-026-77784-7

**Published:** 2026-09-17

**Authors:** Max Hess, Marvin F. Wyss, Edlyn Wu, Gian-Marco Schaniel, Joel Lüthi, Chiara Rebagliati, Daniel Hannuschke, Nadine L. Vastenhouw, Darren Gilmour, Shayan Shamipour, Lucas Pelkmans

**Affiliations:** 1 Department of Molecular Life Sciences, University of Zurich, Winterthurerstrasse 190, Zurich, Switzerland; 2 Systems Biology PhD program, Life Science Zurich Graduate School, University of Zurich, Winterthurerstrasse 190, Zurich, Switzerland; 3 Center for Integrative Genomics, University of Lausanne, Lausanne, Switzerland; 4 The BioVisionCenter, University of Zurich, Winterthurerstrasse 190, Zurich, Switzerland; 5 Molecular Life Sciences PhD program, Life Science Zurich Graduate School, University of Zurich, Winterthurerstrasse 190, Zurich, Switzerland

**Keywords:** 3-D reconstruction, Embryology, Multicellular systems

## Abstract

Multicellular self-organization depends on interactions across multiple length scales, yet mapping protein states at high spatial resolution in whole-mount embryos remains challenging. Here, we introduce high-throughput 3D *in toto* iterative immunofluorescence imaging (3D-4i) and a dedicated computer vision pipeline to quantify morphological and molecular features from subcellular to whole-embryo scales across hundreds of samples. Applying this pipeline to early zebrafish embryos undergoing mid-blastula transition, we determine the cell cycle phase for each cell across the embryo, and uncover the spatiotemporal dynamics by which global meta-synchronous mitotic waves transition to cell cycle desynchronization. Using statistical analysis, we find that the cell cycle phase is the major source of variability in transcription within a division cycle, and combining this with the analysis of key transcription factors and chromatin modifier state, included in our multiplexed dataset, enables accurate prediction of transcriptional output during zygotic genome activation in individual cells. Together, these findings establish 3D-4i as a powerful approach for quantifying multimodal, multiscale biological processes underlying multicellular self-organization.

## Abstract (web summary)

The protein landscapes that underlie multicellular self-organization remain challenging to characterize across intact embryos at high spatial resolution. Here, the authors present 3D-4i, a high-throughput multiplexed imaging and analysis method that maps up to ten protein states across whole zebrafish embryos, revealing how cell cycle and transcriptional activity are coordinated during early development.

## Introduction

A crucial stage in early embryonic development is marked by the transition from maternally provided transcripts to zygotic genome activation (ZGA). In many species, the major wave of ZGA coincides with the lengthening of cell cycles mediated by the introduction of gap phases into the early rapid divisions^1^ (known as mid-blastula transition, MBT). While perturbation studies have identified multiple factors proposed to be causally related to  ZGA - including slowing down of the cell cycle during MBT^2,3^, titration of replication machinery^4,5^, dilution and post-translational modification of maternally supplied histones^6,7^, and the action of pluripotency factors such as Pou5f3 and Nanog^6,8–11^, ZGA is thought to be imprecisely controlled at the single-cell level, raising the question of how these mechanisms contribute to the observed transcriptional variability. Here, using multiplexed embryo profiling, computer vision, and statistical analysis, we investigate the relative contribution of processes underlying ZGA and explore whether its heterogeneous nature reflects the interplay of multiple regulatory processes across different spatiotemporal scales, which collectively ensure a predictable activation of the zygotic genome in individual cells and give rise to reproducible patterns of transcriptional activity in early embryos.

Identifying sources of cell-to-cell variability requires the ability to quantify multiple contributing effects simultaneously in a large number of individual cells within their native spatial context^12–14^. To achieve this in a developmental system, we leveraged the accessibility, abundance, and optical transparency of zebrafish embryos and established a high-throughput approach that combines 3D *in toto* imaging of whole-mount structures with iterative immunofluorescence imaging^15^ (3D-4i). Using this technology, we imaged multiple protein markers across all cells in the entire zebrafish embryo at subcellular resolution. Additionally, we implemented a comprehensive 3D image analysis pipeline to extract features across whole-embryo, single-cell, and subcellular scales for downstream analysis. By applying 3D-4i to a large set of zebrafish embryos undergoing MBT, we capture the multivariate and multiscale nature of processes involved in ZGA at single-cell resolution. Furthermore, we show that these measurements allow the training of models that can accurately predict the transcriptional activity of individual cells during MBT, and identify the position of cells along the cell cycle, in combination with single-cell levels of ZGA-associated factors - particularly Pou5f3, PCNA and H3K27ac - as major predictors of transcriptional output. Finally, we show that restricting cell cycle progression to late interphase using chemical perturbation increased overall transcriptional activity, providing further support for the role of cell cycle control in regulating transcription. Altogether, our results underscore the strength of embryo multiplexing in disentangling the contributions of regulatory factors to complex processes in development.

## Results

### Establishing 3D-4i in zebrafish embryos

**Fig. 1:**
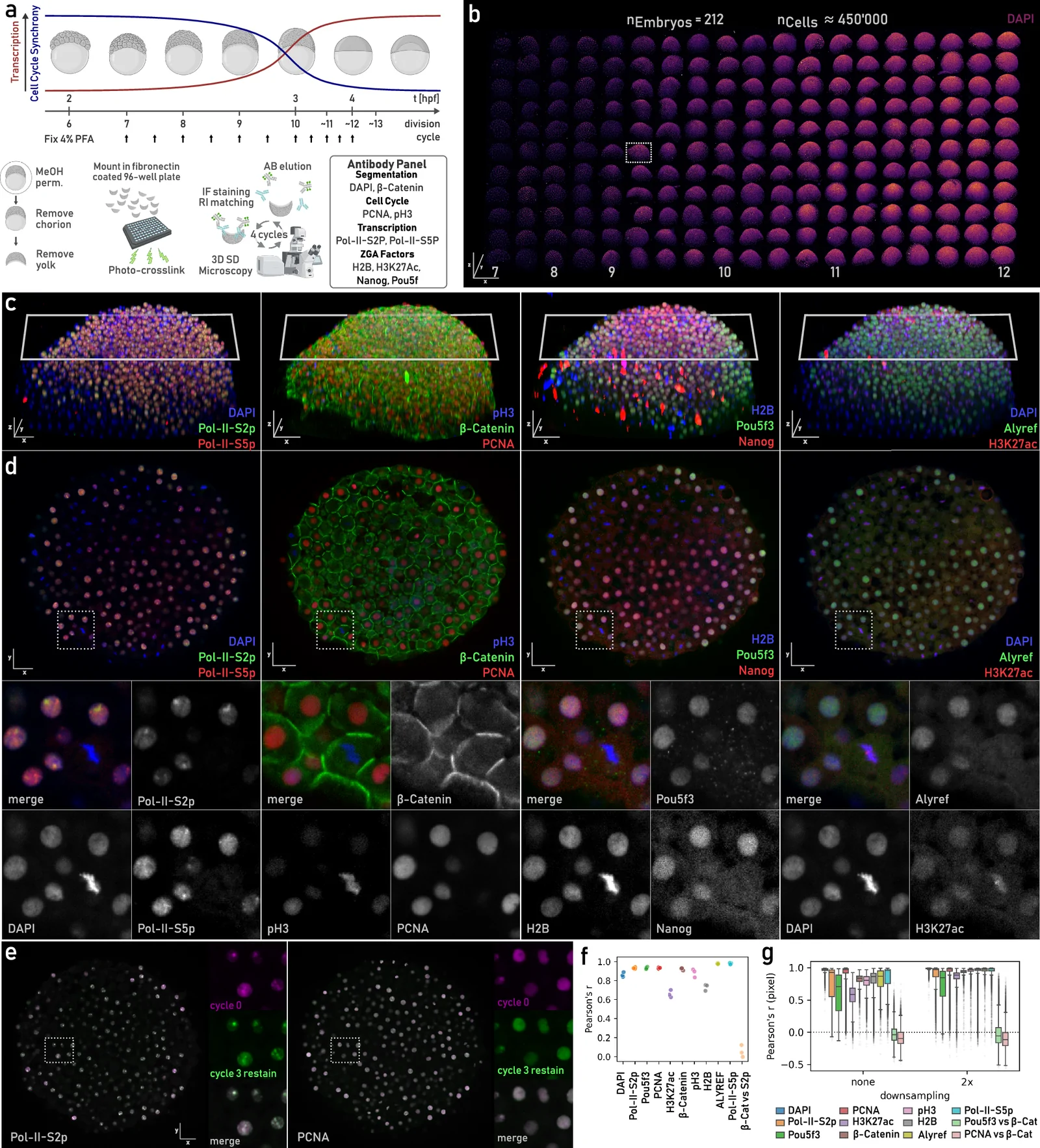
Establishing 3D *in toto* iterative immunofluorescence imaging (3D-4i) in zebrafish embryos. **a** Schematic of zebrafish developmental timepoints used in the multiplexing experiment (top), 3D-4i workflow (bottom left), and antibody panel used in the experiment (bottom right). Created in BioRender. Shami pour, S. (https://BioRender.com/izklq0m) and Inkscape (http://www.inkscape.org). PFA: Paraformaldehyde. hpf: hours post fertilization. MeOH perm: Methanol permeabilization. SD Spinning Disc. RI Refractive Index. IF Immunofluorescence. AB Antibody. For further details, see Methods section. **b** 3D overview of embryos imaged in the multiplexing experiment, ordered from left to right by total nuclei count. Images show the DAPI stain labeling DNA. The ROI demarcates a representative embryo shown in panels (**c**, **d**). Division cycles are marked at the bottom (7-12). *x*, *y*, *z* scale bars 650 µm. *N* = 1 experiment, *n* = 212 embryos (209 shown). **c** 3D view of a representative embryo (demarcated in panel **b**), stained with Pol-II-S2p, Pol-II-S5p, β-Catenin, PCNA, pH3, Pou5f3, H2B, Nanog, Alyref, H3K27ac, and DAPI across four sequential immunofluorescence imaging cycles. The white rectangles demarcate the 2D slice used for visualization in panel (**d**). *x*, *y*, *z* scale bars 50 µm. **d** 2D cross-section of the embryo shown in panel (**c**) along the indicated plane, with zoom-in views shown below. The dashed rectangles demarcate the ROI used for zoom-in. *x*, *y* scale bars 50 µm. *N* = 1 experiment, *n* = 212 embryos. **e** Stainings of Pol-II-S2p and PCNA of an exemplary embryo in acquisition cycles 0 and 3 overlaid on each other, with zoom-in views shown on the right. The dashed rectangles demarcate the ROI used for zoom-in. *x*, *y* scale bars 50 µm. *N* = 1 experiment, *n* = 21 embryos. **f** Pearson’s *r* for mean nuclear intensities (cellular for β-Catenin) between same stains repeated across cycles. β-Catenin (β-Cat) vs Pol-II-S2p (S2p) serves as negative control. **g** Single-pixel correlations between same stains repeated across cycles, at full image resolution (1 pixel: 0.325 µm, left: none) or after 2× down-sampling (1 pixel: 0.650 µm, right, 2x). β-Cat vs PCNA or Pou5f3 serve as negative controls. Center lines: medians; boxes: 25^th^−75^th^ percentiles; whiskers: 1.5× interquartile range; gray dots: outliers; dashed line: zero. For panels **f**, **g** *N* = 1 experiment, *n* = 61 embryos. Source data are provided as a Source Data file.

To establish multiplexed molecular profiling in whole-mount embryos, we adapted the 4i technology, previously applied to mammalian cells grown in culture and 2D tissue sections^13,15,16^, enabling homogeneous immunofluorescence staining, 3D volumetric imaging and efficient antibody elution, thereby facilitating the sequential addition of new antibody sets (3D-4i, Fig. 1a; Methods). A critical step in establishing this workflow was robust sample immobilization, which enabled precise computational alignment of embryos across imaging cycles (Supplementary Fig. 1). To achieve this, we mounted deyolked zebrafish embryos on Fibronectin/Poly-D-Lysine-coated 96-well plates using UV photocrosslinking. This method, combined with a gentle liquid handling system for dispensing and aspirating solutions, minimized embryo displacement and loss, while maintaining embryo morphology during multiplexing cycles. In addition, homogeneous antibody staining of high-abundance targets across the 3D embryo volume was achieved by directly labeling primary antibodies with fluorescently tagged Fab fragments. Finally, embryos were optically cleared using a refractive index-matching solution, allowing volumetric imaging through tissue depths exceeding 250 µm (Methods).

To obtain a dataset for measuring transcriptional activity and its associated factors in individual cells, we performed 3D-4i on 212 early zebrafish embryos, encompassing a total of 458,826 individual cells, collected between 2 and 4 hours post-fertilization (hpf, corresponding to the 7^th^ to 12^th^ division cycles) at 15-min intervals, the developmental time window during which the major wave of ZGA occurs (Fig. 1b). Each embryo was imaged at 20x magnification using automated spinning-disk confocal microscopy across four imaging rounds, generating a volumetric dataset that includes DAPI to quantify DNA content (also used for image registration and nuclear segmentation) and ten-plex protein stainings capturing key molecular markers of the ZGA process. These comprised indicators of cell cycle progression (PCNA, an S phase marker^17^, and phosphorylated H3, pH3, as a marker for mitosis^18^), histone abundance and modifications (H2B and K27-acetylated H3, H3K27ac) reflecting chromatin state, pluripotency transcription factors initiating the zygotic developmental program (Pou5f3 and Nanog), an mRNA nuclear export factor (Alyref), the initiating and elongating forms of RNA Polymerase II (Pol-II-S5p and Pol-II-S2p, respectively) as reporters of transcriptional activity^19^, and finally β-catenin for cell and embryo segmentation (Fig. 1c, d). Notably, due to the lack of a reliable antibody targeting Pou5f3 in zebrafish embryos, we generated a Pou5f3-3xflag transgenic line (Methods) and measured its abundance using anti-FLAG antibody. To minimize technical variability, embryos from different timepoints were pooled and distributed across 30 wells (~7–9 embryos per well).

To assess the reproducibility of staining intensities across imaging cycles, we re-stained markers from the first three cycles in the final cycle, using three replicate wells each, and conducted a correlation analysis between the original and re-stained images (Fig. 1e). Mean nuclear (or cellular for β-catenin) intensities showed high single-cell correlations between the same stains across different imaging cycles and low correlations between unrelated stains (β-catenin and Pol-II-S2p) (Fig. 1f). High single-pixel correlations between the same stains, either in the full resolution or in two times down-sampled images further validated the reproducibility of the stains at the subcellular scale (Fig. 1g). We noticed a slight accumulation of background signal over cycles, resulting in reduced single-pixel correlations of targets with low signal-to-noise ratio (H3K27ac and Pou5f3). Collectively, these results demonstrate that iterative staining in embryos is highly reproducible, compatible with endogenously tagged proteins, and preserves sample integrity also at small length scales, establishing 3D-4i as a high-throughput, whole-mount multiplexed immunofluorescence approach that enables simultaneous investigation of spatiotemporal patterns of multiple protein states in hundreds of zebrafish embryos.

### An image analysis pipeline for multiplexed 3D datasets

**Fig. 2:**
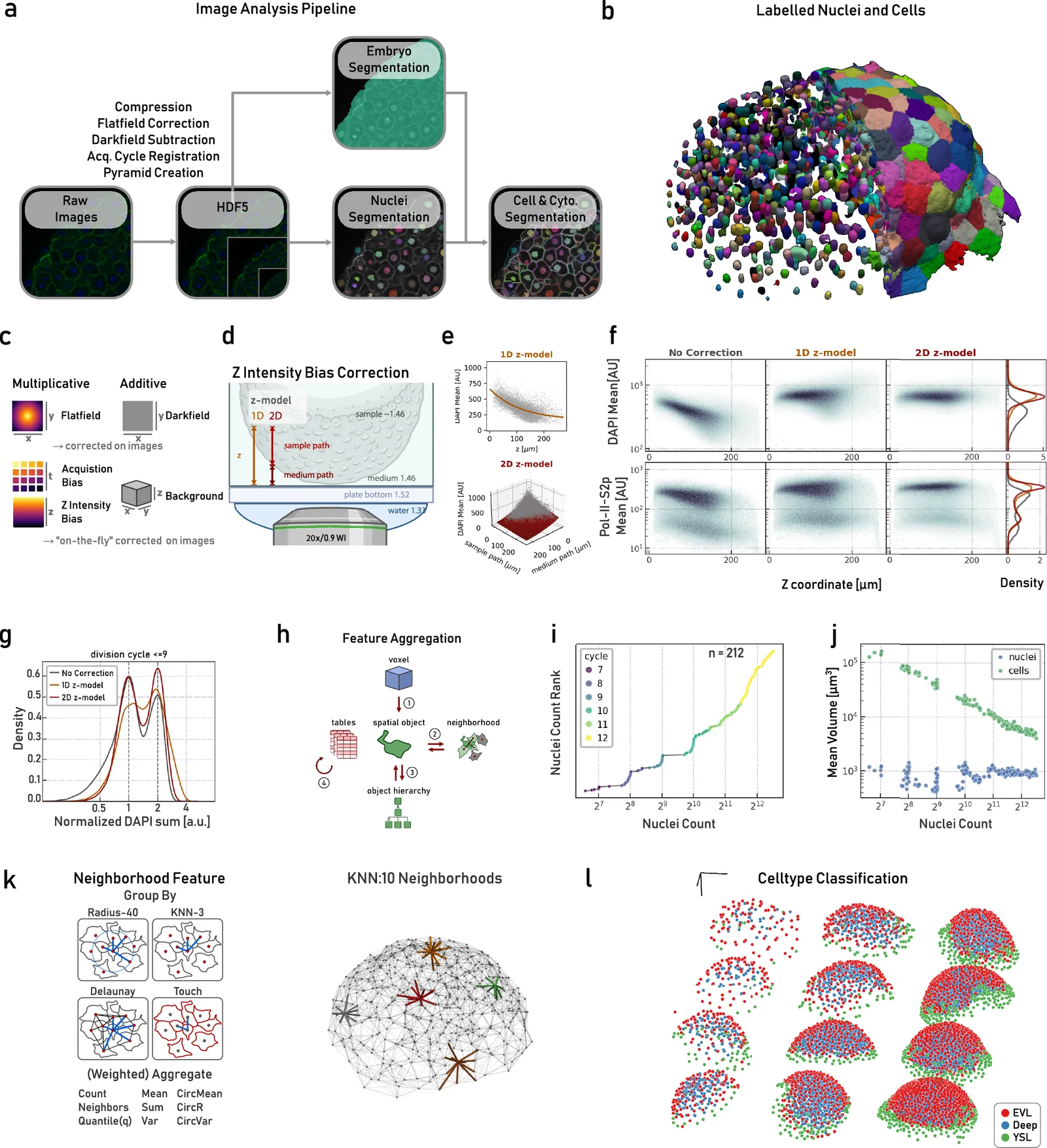
Developing an integrated image analysis pipeline for multiplexed volumetric datasets. **a** Image-analysis pipeline including conversion into multiscale image pyramids, intensity correction, image registration between acquisition (Acq.) cycles and object (embryo, nuclei, cell and cytoplasm) segmentation. **b** 3D view of a representative embryo with nuclear segmentation shown on the left half and cell segmentation on the right half. **c** Series of intensity corrections applied directly to images or corrected on the fly (See “Methods”). Created with Inkscape (http://www.inkscape.org). **d** Schematic of exemplary cell position relative to the objective, showing the light path through the plate bottom, medium, and sample, with their approximate refractive indices. The single-exponential 1D z-decay model only considers the total light path (medium and sample paths sum) and the double-exponential 2D z-decay model considers separate decays for each path. Created in BioRender. Shami pour, S. (https://BioRender.com/izklq0m). **e** Estimation of mean DAPI signal decay using 1D and 2D z-models. **f** Comparison of 1D and 2D z-decay models with uncorrected data for mean DAPI and Pol-II-S2p intensity distributions. **g** The density plot on comparison of 1D and 2D z-decay models with uncorrected data for summed DAPI intensity of embryos in cycles 7–9. Data normalized to the first peak. **h** Logical data model for feature extraction across scales/orders, dividing the process into two steps of label aggregation (across voxels (1), neighborhoods (2), object hierarchy (3) or tables (4)) and computation of summary statistics on aggregated labels. Created with Inkscape (http://www.inkscape.org). **i** Embryo staging based on total nuclear counts. **j** Mean nuclear and cellular volumes plotted against total nuclear counts per embryo. **k** Left: various statistical features (such as mean, sum, etc) can be aggregated across different neighborhoods (fixed radius, touch, *k*-nearest neighbors (KNN) and Delaunay, Methods) to achieve higher scale measurements. Right: Example for KNN neighborhoods with k = 10 in an embryo. Neighbors of exemplary cells shown in colors and thicker lines. Left: Created with Inkscape (http://www.inkscape.org). **l** Results of a random forest classifier trained with the napari feature-classifier plugin to identify three cell types present during early zebrafish development: EVL, YSL and deep cells (Deep). Source data are provided as a Source Data file.

To extract information from these multiplexed volumetric datasets in a systematic and reproducible manner, we next generated a comprehensive image analysis pipeline, encompassing image file compression, multiscale image pyramid generation, intensity correction accounting for various technical aberrations (as detailed below), image registration across staining cycles, and nucleus, cytoplasm, cell and embryo segmentation through the integration of multiple established toolboxes^20–28^ (Fig. 2a–g and Supplementary Fig. 1–2; see “Methods”).

Central to image analysis is to ensure a linear relationship between measured signal intensities and actual fluorophore concentrations. To achieve this, we applied a series of signal corrections either directly to the images or during downstream analysis. First, we applied darkfield and flatfield corrections to the images, which account for thermal noise, camera offset and uneven illumination^28^. Second, we measured background signals by the increase in cytoplasmic signal for each stain, which particularly accumulated in later cycles due to incomplete antibody elution, and corrected this on the extracted intensity features. Third, we estimated for each stain exponential intensity decays caused by fluorophore quenching in the refractive index-matching solution over time (acquisition bias) and along the imaging axis (z-decay bias, Supplementary Fig. 2) due to refractive index mismatches and inhomogeneous scattering in the tissue, and corrected these on the image volumes during post-processing (Fig. 2c; Methods). We used a single exponential model, applied to the mean intensity decay of stains over acquisition time, to account for acquisition bias. For addressing z-decay bias, we tested two models: (a) a single exponential model, which accounts for intensity decay based on the total light path from the objective to the focal plane (1D z-model), and (b) a double exponential model, that separates the light path into the medium and sample distances, accounting for their distinct scattering properties and intensity decay rates (2D z-model) (Fig. 2d). To evaluate the performance of these correction models, we used the mean and sum nuclear DAPI intensity distributions as ground truth. For mean nuclear DAPI intensity, which is expected to be uniform throughout the early embryo, we noted that both z-decay correction models flattened the observed mean intensity decay over the tissue depth, with the 2D z-model further improving the homogeneity of intensity variance along the z-axis. The 2D z-decay model also accounted for intensity decay in other stains, such as Pol-II-S2p, used as a marker for transcriptional output (Fig. 2e, f). For the sum nuclear DAPI intensity, we expect a bimodal distribution, with peaks separated by a factor of two, corresponding to nuclei with two or four DNA copies, based on the cell cycle stage. The 2D model more clearly resolved these peaks and reduced variability around the expected values compared to both the 1D model and uncorrected data, highlighting the necessity and strength of this approach in correcting intensity decays (Fig. 2g and Supplementary Fig. 2).

After intensity corrections and segmentation of spatial objects (e.g., nuclei, cytoplasm, membrane and cells, see “Methods”), we focused on extracting biologically meaningful features from these high-dimensional data comprising ~10^9^ volumetric pixels (voxels) per embryo, across 212 embryos, each with ten-plex protein stainings. To streamline this process, we developed a logical data model that separates feature extraction into two steps: label aggregation and the computation of summary statistical functions on the aggregated labels. In this framework, object measurements such as nuclear volume or mean intensity are acquired by applying statistical functions to aggregated voxels within the same label objects (e.g., nuclei labels). Similarly, we can aggregate features for a given object type, over a series of spatial neighborhoods or across different levels of object hierarchy to derive features at higher length scales/orders. Finally, feature embeddings or cell type classifications can be viewed as aggregating existing features to generate higher-level properties (Fig. 2h). For instance, to identify the developmental stage of embryos, we can aggregate nuclei objects at the embryo hierarchy level and sum their total counts. As expected, we find that, up to the 10^th^ cell cycle, the nuclei count clustered around powers of two, reflecting the rapid and meta-synchronous nature of early embryonic division cycles. However, as synchronicity is lost in later cycles^17,29^, a more gradual increase in nuclei count between embryos is observed (Fig. 2i). Furthermore, plotting mean nuclear and cellular volumes against the number of nuclei per embryo revealed a halving of cell volume with each division cycle, while nuclei volume remained relatively constant (Fig. 2j). Similarly, local cell density in the embryos can be measured, for instance by aggregating cell count within a 50 μm radius neighborhood, revealing that density varies locally, and is typically higher in the center than in the periphery of the embryo (Fig. 2k and Supplementary Fig. 3a). Leveraging this logical data model, features such as position, nuclear morphology and mean intensity of stains were aggregated to train a random forest classifier that distinguishes fluorescent debris from actual nuclei and enables unbiased identification of the three cell types observed during early zebrafish development: the enveloping layer cells (EVL), yolk syncytial layer (YSL), and deep cells giving rise to embryo proper (Fig. 2l and Supplementary Fig. 3b; Methods). The classifier’s predictions were then used to remove debris and exclude YSL cells from downstream analysis, as they often exhibit distinct nuclear morphologies in comparison to deep and EVL cells^30^. Collectively, this logical data model serves as a unifying framework for feature aggregation, enabling the integration of new features and aggregation modes. Together with the developed image analysis pipeline, this supports robust measurements across multiple spatial and hierarchical scales.

### Multiplexed imaging reveals cell cycle asynchrony during MBT

Cell cycle phase (CCP) is a known source of variability in transcriptional activity during ZGA^1–3,10,31,32^, thus we developed methods to reliably infer CCP in fixed samples, enabling analysis of the impact of early cell cycles on transcriptional output. Since many cellular and nuclear features change throughout CCP, we hypothesized that a randomly selected population of cells should organize along a circular manifold in a high-dimensional feature space defined by cell cycle-related attributes such as nuclei morphology, DAPI and PCNA intensities^33^. This manifold should allow the estimation of each cell’s position along the cell cycle trajectory, providing a systematic approach for the analysis of cell cycle dynamics from fixed images.

**Fig. 3:**
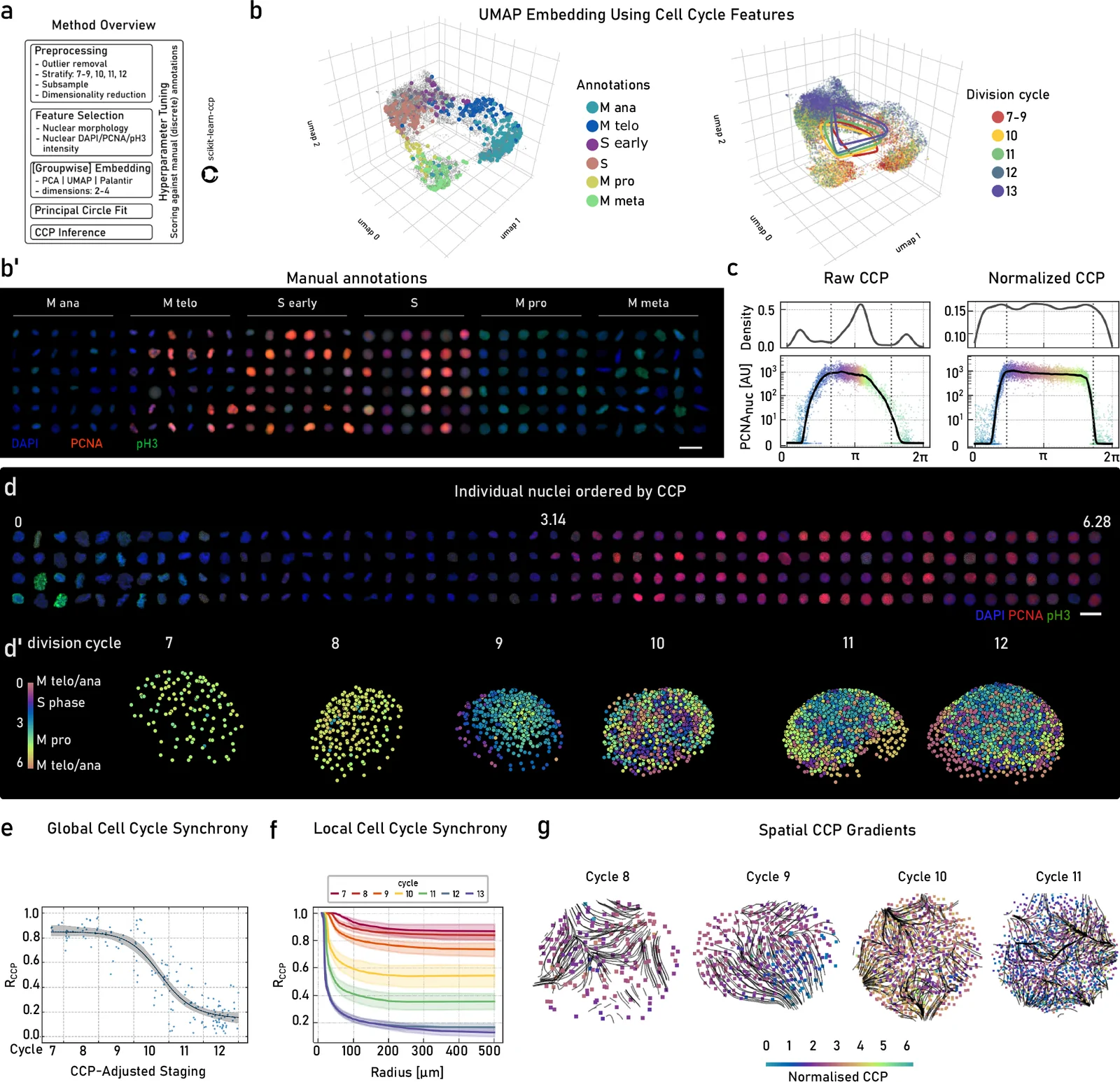
Inferring cell cycle phase from multiplexed imaging of fixed embryos reveals spatiotemporal dynamics of cell cycle desynchronization during mid-blastula transition. **a** Method overview for pre-processing, single-cell embedding into a low-dimensional space using cell cycle-related features, principal circle fitting in feature space and CCP inference (Methods). **b** Left: UMAP embedding of cell cycle features overlaid with manual cell cycle annotations. Right: Principal circles fitted to feature space of cells from division cycles 7–9, 10, 11, 12, and 13 separately. S S phase, M mitosis, pro prophase, meta metaphase, ana anaphase, telo telophase. **b’** Representative nuclei annotations for six cell cycle phases visualized using DAPI, PCNA, and pH3 markers. Scale bar 20 μm. **c** Density plot and mean nuclear PCNA intensity of 10,000 cells randomly sampled across division cycle 11, plotted against raw CCP (left) or normalized CCP (right, hereafter CCP) after inference and quantile-based normalization. Points are colored by normalized CCP, illustrating the monotonic mapping introduced by normalization. Black line: LOWESS fit highlighting observed dynamics. Dotted vertical lines correspond to M anaphase/telophase, S, and M pro/meta phases. **d** Exemplary nuclei ordered by inferred CCP and visualized with DAPI, PCNA, and pH3 markers. Scale bar 20 μm. **d’** Representative embryos from division cycles 7 to 12 with nuclei shown as point clouds and colored by their inferred CCP. **e** Global cell cycle synchrony (*R*) plotted against CCP-adjusted staging, fitted with a Hill function (Methods). The solid line is the bootstrap-mean fit, and the shaded region shows the pointwise 95% bootstrapped confidence interval. **f** CCP synchrony measured over increasing radii across division cycles 7–13. Solid lines indicate group medians for the corresponding division cycle, and the shaded regions mark 95% bootstrapped confidence intervals. **g** Spatial CCP gradients for 4 representative embryos from division cycles 8 to 11. Streamlines correspond to the integrated gradient field starting from 500 randomly sampled sources within the convex hull of nuclei positions. Nuclei colors denote the inferred CCP. Source data are provided as a Source Data file.

To this end, cells were embedded in lower-dimensional spaces using Uniform Manifold Approximation and Projection (UMAP^34^), following pre-processing steps such as data subsampling to address class imbalances across division cycles and feature selection/normalizations (Fig. 3a; Methods). As expected, cells pooled from all timepoints organize along a circular manifold in a 3-dimensional UMAP embedding of cell cycle features. While the first two UMAP components capture cell cycle progression, the third axis largely corresponds with changes related to consecutive division cycles (Fig. 3b). A principal circle was then fitted to the embedded nuclei of each division cycle to model the continuous and circular nature of cell cycle progression and correctly map 634 manually annotated nuclei to their corresponding cell cycle phase (Fig. 3b, b’; Methods). The CCP value for each cell, which we bounded between 0 and 2π, was then defined from the closest point on the principal circle of its respective division cycle (Methods). Finally, because distinct cell cycle phases have different durations, resulting in a non-uniform distribution of cells along the cell cycle (Fig. 3b), we normalized the CCP density (hereafter termed CCP), resulting in a uniform distribution that reflects the progression of cells through the cell cycle (Fig. 3c). To validate this inference approach, we performed live imaging of embryos injected at the one-cell stage with histone H1 conjugated to Alexa Fluor 488^35^, tracked nuclei throughout a division cycle and quantified the corresponding dynamics of effective nuclear radius. We find that nuclear morphology dynamics of in vivo-tracked nuclei closely matched the inferred dynamics estimated from our fixed multiplexed dataset, supporting the accuracy of the CCP inference model in ordering the cells along a pseudotemporal axis (Supplementary Fig. 4a, a’).

**Fig. 4:**
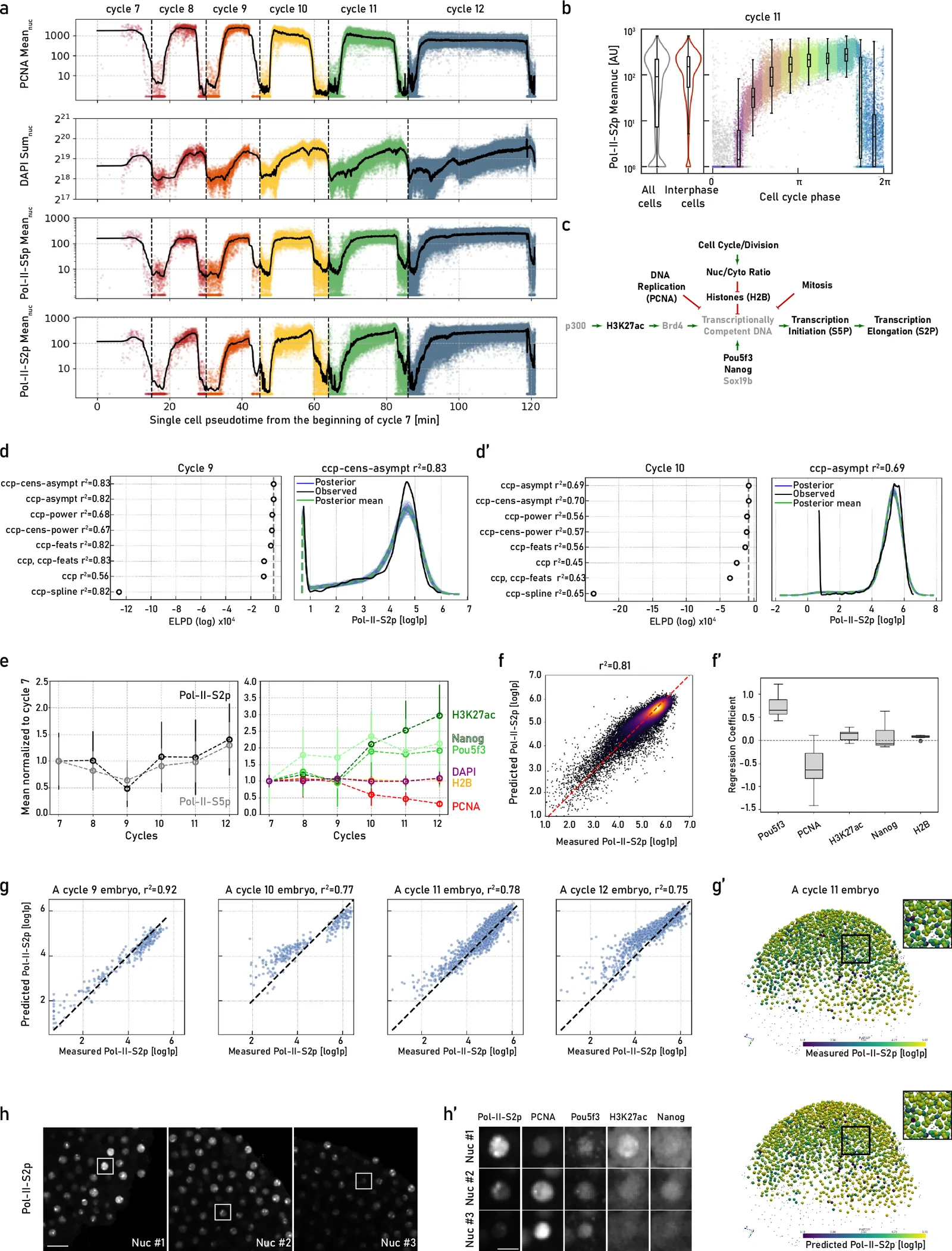
Multiplexed embryo profiling links cellular state to zygotic genome activation in single cells. **a** Mean PCNA, Pol-II-S5p and Pol-II-S2p, and sum DAPI intensities across division cycles 7–12. Solid lines. Gaussian-smoothed trends; dashed lines and color changes, cycle transitions. **b** Left: Mean nuclear Pol-II-S2p intensities of all (gray) and interphase (red) cells during division cycle 11. Right: Same data plotted against their inferred CCP. Cells were grouped into nine CCP bins (six interphase and three mitotic), shown by distinct colors with overlaid boxplots. **c** Known interactions controlling transcriptional output^10,76^. Green/red arrows indicate activating/repressive interactions. Gray components represent known regulators not measured here. **d**–**d’**. Left: Comparison of models using CCP or CCP-inference features as linear or non-linear predictors of transcriptional output. Models are ranked by held-out predictive performance using expected log pointwise predictive density (ELPD) and *R*². asympt: asymptotic; ccp-feats CCP features; cens censored. Right: Posterior predictive distributions of the best-performing model compared with observed data. Panels d/d’ show cycles 9/10, respectively. **e** Mean interphase dynamics of Pol-II-S5p, Pol-II-S2p, H3K27ac, Pou5f3, Nanog, H2B, PCNA and DAPI for deep cells across division cycles 7-12. Error bars, standard deviation. **f** Measured versus predicted Pol-II-S2p levels for interphase cells with CCP held constant. S phase was divided into six equal strata, and multilinear regression was applied within each bin. Colors indicate density; the red line marks equality. **f’** Regression coefficients for each measured ZGA-associated factor across interphase bins. Gray circle: outlier; dashed line: zero. **g** Measured versus predicted Pol-II-S2p levels for four held-out embryos using the best-performing cycle-specific models. Black lines indicate equality. **g’** Measured and predicted mean nuclear Pol-II-S2p in a cycle 11 embryo. Excluded nuclei and artifacts are shown in gray; boxes indicate zoom-ins. **h** Representative cycle 11 embryo stained for Pol-II-S2p. White boxes indicate the exemplary nuclei (nuc) shown in (**h’**). **h’** Exemplary nuclei, indicated in h, stained with Pol-II-S2p, PCNA, Pou5f3, H3K27ac and Nanog antibodies. Scale bars 25 µm in h and 10 µm in (**h’**). In **b** and **f’**, center lines: medians; boxes: 25^th^−75^th^ percentiles; whiskers: 1.5× interquartile range. Data in this figure is the same as in Fig.1: *N* = 1 experiment, *n* = 212 embryos. Source data are provided as a Source Data file.

Having inferred the CCP values for all cells within each embryo (excluding YSL nuclei), we refined embryo staging based on the circular mean CCP in combination with nuclear count (CCP-adjusted staging, Supplementary Fig. 4b; Methods). When visualizing the CCP of all cells in their spatial contexts, we observed that in early embryos (division cycles 7 to 9), cells exhibit similar CCP values (meta-synchronous), whereas with increasing developmental time, the cell cycle becomes asynchronous across the embryo (division cycles 10-12, Fig. 3d, d’). To quantify the dynamics of the transition from meta-synchrony to asynchrony, we measured the circular synchrony of the CCP (R) for all cells within each embryo (Methods), where *R* = 1 indicates perfect synchrony, and *R* = 0 corresponds to complete asynchrony. This analysis revealed that up to cycle 10, average CCP synchrony remains high (above 0.7), but drops sharply at cycle 10 (fitted Hill coefficient of ≈ 22; Methods) and approaches near-complete asynchrony ( ~ 0.1) at cycles 11 and 12 (Fig. 3e). This abrupt increase in CCP heterogeneity at division cycle 10 coincided with a switch-like rise in transcriptional activity, as indicated by measurements of Pol-II-S2p foci volume (Supplementary Fig. 5), which carry the majority of nascent RNAs^3^, pointing at the potential causal link between CCP dynamics and zygotic genome activation (see Fig. 4). In addition, we analyzed whether there is a spatial component to cell cycle desynchronization by quantifying CCP synchrony between cells positioned at increasing distance from each other (from 0 to 500 μm reaching the embryo boundaries). This analysis demonstrated a half maximum synchrony at ~100 μm in cycle 7 embryos that steadily decreased to ~25 μm by cycles 12 and 13, corresponding to ~ one cell diameter (Fig. 3f), suggesting that a gradual decrease in spatial synchrony might underlie the observed rapid temporal transition in global CCP synchrony. In line with this, calculating spatial gradients of mitotic progression using the inferred CCP values (Methods) revealed coherent pseudo-waves in cycles 8 and 9, and more chaotic local patterns in cycles 10–11 (Fig. 3g), as previously reported^17,29^. Altogether, by integrating machine learning algorithms and statistical approaches, we inferred CCP at the single-cell resolution, enabling quantification of the spatiotemporal dynamics of cell cycle transitions from meta-synchrony to asynchrony and establishing the foundation for assessing the role of CCP in regulating transcriptional activity.

### Multiplexed embryo profiling links cellular state to ZGA

We next investigated whether spatially resolved measurements that capture multiple features across biological scales provide contextual information that explains the heterogeneity observed in transcriptional activity of individual cells during zygotic genome activation. Because CCP is a major source of variability in cellular states of mammalian cells grown in culture^15^ and becomes abruptly asynchronous in division cycle 10 embryos coinciding with ZGA onset (Fig. 3e and Supplementary Fig. 5), we visualized variation of the measured features in our multiplexing experiment across CCP. To this end, we generated a pseudotemporal axis of cell cycle progression from division cycle 7–12 by mapping individual cells onto developmental time, using their embryonic stage and inferred CCP scaled by division cycle durations obtained from live-imaging experiments^29^. Examining DAPI sum intensity demonstrated that DNA content dropped immediately after chromosome segregation and doubled during S phase, while mean PCNA intensity showed rapid transitions between high values in S phase and low values in M phase, as expected^17^ (Fig. 4a and Supplementary Fig. 6a). Plotting mean nuclear intensity of Pol-II-S5p and Pol-II-S2p along this axis as a proxy for average transcriptional activity revealed two types of dynamics: A slow, gradual increase across successive division cycles, as well as variations within each cell cycle, characterized by low activity of RNA Pol-II during mitosis followed by a sharp increase in activity during S phase, eventually reaching a plateau (Fig. 4a). As expected, transcription initiation, marked by Pol-II-S5p, preceded transcription elongation, measured by Pol-II-S2p (Supplementary Fig. 6a’) and inhibiting RNA Pol-II with triptolide led to decrease in transcription levels (Supplementary Fig. 7a, a’; Methods). Consistent with its cell cycle-dependent dynamics, variation in transcriptional activity between individual cells of a given division cycle was markedly reduced when considering interphase cells only or cells belonging to similar CCP (Fig. 4b). To investigate the statistical association between CCP and transcriptional output, we implemented a Bayesian regression framework to predict zygotic transcriptional activity in single cells, as measured by mean nuclear Pol-II-S2p during interphase, using the inferred CCP of each cell with either linear or non-linear functions (Methods). Model performance was assessed by explained variance (*R*²) and prediction accuracy was evaluated on held-out data, revealing that the relationship was best described by a non-linear asymptotic regression model (ccp-asympt, Fig. 4d–d’ and Supplementary Fig. 6b). Using our modeling framework, we found that CCP alone is a strong predictor of transcriptional activity, explaining between 65–85% of the variance across individual cells in division cycles 8–12 (Fig. 4d–d’ and Supplementary Fig. 6b)^32^.

To study the variance in transcriptional activity not explained by CCP, we next quantified the abundance of the proteins associated with ZGA measured in our multiplexed dataset (Fig. 4c). At the embryo-averaged level, we observed a pronounced decrease in PCNA as division cycles progressed, concomitant with a rise in transcription initiation and elongation, consistent with previous models proposing that titration of replication machinery is required for transcriptional activity^4,5,36^ (Fig. 4e). In contrast, the pluripotency transcription factors Nanog and Pou5f3, together with the active chromatin mark H3K27ac increased markedly from division cycle 10 onwards (Fig. 4e), indicating that the combined chromatin and transcription factor landscape progressively acquires transcriptional competence during MBT^6–8,37^. Interestingly, EVL cells displayed similar trends in these measurements, albeit displaying slightly higher transcriptional activity compared to deep cells, in line with their early differentiation and epidermal function in the embryo^38^ (Supplementary Fig. 6c). Importantly, at the single-cell level, these markers displayed substantial heterogeneity, which paralleled the increasing heterogeneity in transcriptional output observed during later cycles (Fig. 4e and Supplementary Fig. 5a’). To disentangle CCP-dependent from CCP-independent contributions to transcription, we performed multilinear regression modeling using ZGA factors (Pou5f3, Nanog, PCNA and H3K27ac together with H2B, as histones have been shown to compete with these transcription factors for DNA binding^6^) across narrow interphase time windows, where mean PCNA remained stable (Methods). This analysis revealed that ZGA-associated factors account for a substantial proportion of the residual transcriptional variability independent of CCP (Fig. 4f). The quantified relative contribution of these factors in predicting transcriptional output and permutation analysis showed a strong contribution from Pou5f3, followed by PCNA, H3K27ac and Nanog (Fig. 4f’ and Supplementary Fig. 6d). Finally, we built a comprehensive model that combines the non-linear CCP relationship with additional contributions from ZGA factors, CCP-related intensity features and morphological properties such as nuclear size and nucleus/cytoplasmic volume ratio as linear predictors (Methods). These models generalized across held-out embryos (i.e., not used for model training) and more accurately predicted Pol-II-S2p levels compared to models only considering CCP or not including the nonlinear effects of CCP (Fig. 4g, g’ and Supplementary Fig. 6e, f), even on spatially proximate cells within the same embryo that displayed strikingly different transcriptional activities (Fig. 4h, h’). Thus, by leveraging quantitative, image-based measurements of multiple markers that integrate these processes across scales, our model provides a predictive framework for how individual cells in the zebrafish embryo transition from maternal to zygotic genome activation.

Finally, to test the predictions of our modeling approach regarding the role of CCP in transcriptional regulation, we measured transcriptional activity in embryos treated with cell division-blocking cocktail HUA (hydroxyurea and aphidicolin)^39,40^, which restricts cell cycle progression to S phase. We find that HUA-treated cells were shifted toward the late S phase in the cell cycle, displaying higher PCNA marker, and, consistent with the predictions of our statistical model, exhibited higher transcriptional activity compared to control cells undergoing normal cell division (Supplementary Fig. 7b, b’). Together, these results demonstrate that transcriptional heterogeneity during ZGA in early zebrafish embryos at the single-cell level reflects both within-cycle variations shaped by CCP and across-cycle accumulation of pluripotency factors and chromatin activation marks.

## Discussion

We here extended iterative indirect immunofluorescence imaging (4i)^15^ to confocal *in toto* imaging of early zebrafish embryos (3D-4i), and collected a ten-plex protein dataset in 212 embryos of more than 250 µm in thickness each, resulting in multiplex measurements for ~10^11^ volumetric pixels. To analyze such terabyte-scale datasets, we built an integrated image analysis pipeline encompassing image pre-processing, nuclei, cell, and embryo segmentation, as well as multiscale feature extraction. Combined with time-course experiments, this offers a comprehensive platform for identifying properties that emerge at different length and time scales as revealed by the presence, abundance, and spatial organization of multiple proteins and protein states in complex 3D systems. By applying this platform to ZGA in early zebrafish embryos, a hallmark developmental transition that is highly heterogeneous between individual cells, we show that it allows the collection of measurements that can train predictive models with high single-cell precision on held-out datasets. This provides a foundation for future machine learning-driven approaches to model cellular decision-making in 3D multicellular systems that are both predictive and mechanistically insightful, when combined with perturbation experiments.

ZGA is orchestrated by multiple molecular processes, including cell cycle progression, chromatin state and the nuclear abundance of pluripotency factors that collectively shape the nuclear and transcriptional landscape of individual cells. Despite much progress in understanding the mechanisms that drive the complex interactions of these factors within individual cells, it has remained unclear what causes the heterogeneous nature of ZGA onset across embryos. By simultaneously measuring cell cycle markers, ZGA-associated factors and transcriptional activity at subcellular resolution across a large number of embryos, we demonstrate that i) cell cycle phase (CCP) can be reliably inferred for every cell within the embryo from fixed samples; ii) CCP is the principal source of variability in zygotic transcription; and that iii) CCP, together with ZGA-associated factors, accurately predict transcriptional heterogeneity at single-cell level. The non-linear relationship between CCP and transcriptional activity likely reflects constraints imposed by the DNA replication machinery on chromatin accessibility for transcription, while the additional contribution of ZGA-associated factors underscores their role in modulating transcriptional competence within cell cycle-defined phases. Altogether, these findings suggest that the observed heterogeneity may not arise from imprecise control, but instead could demonstrate the multimodal nature of processes acting at different temporal scales. Recent findings in zebrafish and Xenopus embryos indicate that variation in cell cycle duration across the embryo contributes to differences in the timing of ZGA onset^17,41^. Consistent with this, our pharmacological perturbation of the cell cycle, which blocked cells in the S phase of the 10^th^ division cycle, led to an increase in transcriptional activity. By contrast, earlier studies in fruit fly and zebrafish embryos suggested that cell cycle lengthening alone is insufficient to prematurely trigger ZGA^18,42^. Our statistical framework reconciles these observations by supporting a multivariate model of transcription, in which CCP heterogeneity within a given division cycle contributes to variation in ZGA timing, while additional factors that change progressively across division cycles further shape the transcriptional landscape. Future studies will be required to mechanistically dissect these effects by perturbing both cell cycle-dependent and cell cycle-independent processes and examining their combined influence on transcriptional heterogeneity and ZGA onset.

The observed heterogeneity in transcriptional output and the timing of ZGA onset raises the question of whether this variability contributes to later patterning events after the mid-blastula transition. Cell-to-cell heterogeneity has been shown to facilitate lineage decisions and patterning during multicellular self-organization^43–45^. For example, variation in cell cycle length in *Dictyostelium* generates heterogeneous cell cycle states at the onset of patterning, thereby producing diverse responses to differentiation-inducing signals and supporting robust, reproducible fate decisions under fluctuating extrinsic conditions^46^. Conversely, extreme variability in cellular responses to signaling are corrected during development^47,48^, suggesting that excessive heterogeneity can be detrimental. Whether variability in cellular properties is actively controlled during embryogenesis, and whether it influences how individual cells perceive signaling cues during symmetry breaking, remains to be determined.

While the current study focuses on the molecular processes underlying ZGA, 3D-4i is broadly applicable to multivariate processes in development, regeneration, and disease. Cellular behaviors such as shape changes, migration, proliferation, and fate specification can be viewed as outcomes of multimodal processes that operate across scales. These decisions are often regulated by a combination of external signals and intrinsic physicochemical properties of cells^13,49–51^, yet how these inputs are integrated to drive distinct spatiotemporal gene expression programs remains poorly understood. Provided that reliable antibodies are available, 3D-4i can enable the simultaneous mapping of multiple subcellular processes, including signaling activity, chromatin and transcriptional changes, cytoskeletal remodeling, the endomembrane system, and the translational machinery, thereby providing a holistic framework to disentangle their relative contributions to cellular behavior, tissue patterning, and organismal development.

Although we analyzed a large number of samples, achieving near-continuous coverage of rapid processes such as the early division cycles remains challenging, as illustrated by the incomplete representation of the 9^th^ division cycle in our dataset (Supplementary Fig. 4b). To identify and account for such under-sampling artifacts, detailed estimation of embryo age based on nuclear count or morphology may provide a useful strategy. In addition, high-dimensional cellular measurements could be used to link cells across developmental time points using approaches such as optimal transport^52^, thereby reconstructing pseudodynamic trajectories of developmental outcomes at single-cell resolution.

Importantly, even with the modest number of proteins and protein states included in our multiplexing panel, the multivariate set of quantitative features extracted or derived from these stains achieved high prediction accuracy. This finding demonstrates that, with careful marker selection and explicit consideration of confounding biological processes, such as the cell cycle, highly predictive computational models can be derived even with limited molecular inputs. Nevertheless, further optimization of multiplexed imaging technologies will be essential to expand the number of detectable proteins and post-translational states. Future developments may benefit from complementary preservation strategies, including stronger fixation or embedding methods, as well as improved elution and de-lipidation protocols, to completely abolish background signal accumulation. Ultimately, integrating such higher-plex protein state imaging with multiplexed fluorescent in situ hybridization (FISH)^53^ and single-cell multi-omics data, including chromatin accessibility and transcriptomics^38,54,55^, will be necessary to enable the construction of comprehensive systems-level models that capture the flow of information across spatial and temporal scales that drive pattern emergence during early development.

### Methods: Fish line maintenance and embryo staging

Zebrafish (*Danio rerio*) were maintained in the fish facility of Prof. Darren Gilmour. All animal work was carried out in accordance with the FELASA guidelines and standards of the University of Zurich. Embryos were raised at 28 °C in E3 medium until reaching the corresponding developmental stage. During sample collection, initial embryo staging was performed according to time post fertilization^56^. The experiments were carried out on Pou5f3-3xflag transgenic line, which was generated by Dr. Edlyn Wu in the laboratory of Prof. Nadine Vastenhouw at the University of Lausanne according to Swiss regulations (canton Vaud, license number VD-H28).

### Generation of *pou5f3:3xflag* zebrafish line

The 3xflag knock-in *pou5f3* line was generated using CRISPR/Cpf1^57^. Guide RNA (gRNA) was designed using CHOPCHOP tool^58^ and targets a sequence close to the C-terminal end of *pou5f3­* gene 18 bp upstream of the stop codon. The sequence of gRNA is as follows (with PAM sequence underlined): TTTGGTGGGTCATCTCACCAGCTAACTT. The sequence of DNA repair template (donor ssODN) is as follows (with mutated PAM sequence underlined, 3xFLAG sequence in brackets, and stop codon in bold): TGCCATCCCTCCACCGACCAGATGTCTTCAAAAACGGCTTGCACCCTGGTCTAGTGGGTCATCTCACCAGC[GACTACAAAGACCATGACGGTGATTATAAAGATCATGATATCGATTACAAGGATGACGATGACAAG]**TAA**CTTGCTCCAGGTGCTCACTAAAGTTTATAGAAA (173 nts, synthesized by IDT). RNP mix was first prepared as follows: 1 μl EnGen LbaCas12a (Cpf1) protein (M0653T, NEB); 0.353 μl gRNA (at 100 μM stock), 5 μl RNP buffer (50 mM KCl, 2.5 mM MgCl_2_, 5 mM Tris-HCl pH 8), and 1.65 μl nuclease-free water. RNP mix was incubated at 37 °C for 10 min and then kept at room temperature before use. To prepare Cpf1 injection mix, 4 μl RNP mix was used and 0.5 μl 400 ng/μl ssODN and 0.01% phenol red were added. A final amount of 150 pg gRNA, 40 pg ssODN, and 20 fmol Cpf1 was injected into the one-cell stage of zebrafish embryos. Injected embryos were grown for three months at 28 °C before screening for mutant founders. Founders were outcrossed to wild-type (TL/AB) fish. To obtain homozygous fish, outcrossed fish originating from two founders were in-crossed. Homozygous fish were screened by fin clipping. Homozygous *pou5f3-3xflag* embryos were produced by in-crossing identified homozygous fish.

### Sample preparation

*pou5f3-3xflag e*mbryos were collected in 15-min intervals between 2 and 4 h post-fertilization (hpf) and fixed in fresh and pre-cooled 4% paraformaldehyde (PFA; Lucerna Chem) in 1X PBS. Tubes were kept on ice during sample collection and were then transferred to a rotary shaker at 4 °C for overnight fixation (up to 18 h). Fixation was halted by thoroughly washing the embryos five times with 1X PBS, followed by quenching with 100 mM ammonium chloride (Sigma-Aldrich) for 30 min. Embryos were then dechorionated in a glass Petri dish using watchmaker forceps under a stereo microscope and permeabilized by gradually increasing the methanol concentration in 25% increments up to 100% (Sigma-Aldrich), with 5-min intervals between each step. The embryos were then incubated in 100% methanol at −20 °C for 2 days. Successful storage was tested for up to 3 months. On the experiment day, samples were re-hydrated back to PBS-T (PBS + 0.1% Tween 20; Sigma-Aldrich) in 25% steps. Consequently, the yolk was removed by agitating the samples in a glass centrifuge tube using a Pasteur pipette in at least six rounds of washing with PBS-T.

### Mounting on 96-well plates

Plate preparation was done on the day of mounting. The wells of a polystyrene, flat-bottom 96-well plate (Greiner) were coated as follows to facilitate sample adherence. The coating solution consisted of a 1:10 dilution of human fibronectin (Sigma-Aldrich) in PBS mixed with a 1:2 dilution of Poly-D-Lysine (Thermo Fisher) in PBS. Each well received 50 μl of the solution and was incubated for  2 h at room temperature. After incubation, the liquid was aspirated, and the plate was left to dry for an additional 2 h. Wells were filled with 300 μl PBS, then the embryos were carefully transferred into the wells using a glass Pasteur pipette with a Pipet Pump II (Sigma-Aldrich) to enable gentle handling. Allowing the embryos to settle naturally within the liquid column of the filled well ensured their robust orientation with the animal pole facing the bottom of the plate. Up to 9 embryos were mounted in a single well to maintain proper spacing between them for imaging. Embryos from different time points were pooled and randomly distributed within wells to alleviate potential sources of technical bias between different developmental timepoints. After mounting, the plate was incubated for  2 h before centrifugation at 200 rcf for  5 min on a plate centrifuge. To enhance adherence, the plate was placed on a UV transilluminator (Lab Gene Instruments) for  1 h at 365 nm with high intensity for crosslinking. It was then stored at 4 °C overnight before proceeding with the next steps. For additional crosslinking, an imaging session was performed on the Cell Voyager 8000 microscope, acquiring a 100 μm stack with a z-spacing of 4 μm using the 488 nm laser at 100% laser power and 100 ms exposure.

### Whole-mount multiplexed immunofluorescence

The protocol for performing 3D-4i on zebrafish embryos was devised based on standard immunofluorescence protocol for zebrafish oocytes/embryos^59,60^, the original 4i method^15^ and whole-mount volumetric imaging^61^. Embryos were prepared and mounted in a 96-well plate as described before. Each cycle of the multiplexing experiment consisted of the following steps, all conducted at room temperature:

1. Antigen Retrieval/Antibody Elution
2. Blocking
3. Primary Antibody Incubation
4. Secondary Antibody Incubation
5. Optical Clearing by Refractive Index (RI) Matching
6. Imaging
7. RI Matching Buffer Washout

All pipetting steps were executed using the Bravo automated liquid handling platform (Agilent) with protocols described below. A residual volume of 70 μl was always maintained in each well to avoid sample disturbance when aspirating/dispensing liquid.

1. Antigen Retrieval/Antibody Elution The 3D-4i elution buffer (similar to 4i elution buffer: 0.5 M L-Glycine (Sigma-Aldrich), 1.2 M Urea (Sigma-Aldrich), 3 M Guanidinium chloride (Sigma-Aldrich)) was prepared in advance, with Tris(2-carboxyethyl)phosphine hydrochloride (TCEP; Sigma-Aldrich, 0.07 M) added just before use. The pH was then adjusted to 3 using 32% HCl (Sigma-Aldrich). The buffer was introduced via multiple volume replacement and mixing steps. After incubating for 1 h, wells were thoroughly washed 12 times with PBS (total duration of 30 min), as such pH returned to ~7.

2. Blocking The 3D-4i blocking buffer (PBS with 1% BSA and 0.2 M Ammonium chloride; Sigma-Aldrich) was prepared at 2X concentration in advance. Before use, Maleimide (0.3 M; Sigma-Aldrich) was added. The blocking buffer was mixed with an equal volume of residual PBS in the wells and embryos were incubated in the blocking buffer for 1 h, followed by extensive washing with PBS.

3. Primary Antibody Staining 3.1 Indirect Immunofluorescence: Primary antibodies were prepared at 2X concentration in a 2X 3D-4i blocking buffer (without Maleimide) and mixed with an equal volume of residual PBS in each well. Samples were incubated in this solution overnight at room temperature on a gentle see-saw rocking device.

3.2 Direct Immunofluorescence: Antibodies against high-abundance targets showed strong staining bias from periphery to the center of the sample^62^. To alleviate this issue, these primary antibodies were directly labeled with fluorescently tagged Fab fragments. The ZenonTM IgG labeling kits (Invitrogen) were used as indicated by the manufacturer’s protocol. In short, 1 μg of primary antibody was prepared and incubated with 5 μl of Zenon Fab fragments for 5 min. Surplus unbound Fab fragments were blocked using 5 μl of corresponding species blocking buffer provided in the kit, incubating for another 5 min. Labeled primary antibodies were prepared fresh for each staining and incubated overnight with embryos at room temperature. No additional secondary step was performed, except for DAPI staining prior to imaging. List and dilution factors for each primary antibody and the cycle in which they were stained, are listed in Supplementary Table 1. We note that Anti-FLAG antibody staining in *pou5f3-3xflag* embryos serves as a readout for Pou5f3 protein abundance.

4. Secondary Antibody Staining

Excess primary antibodies were removed by extensive washing with PBS. Secondary antibodies were diluted at 2X concentration in 3D-4i blocking buffer (without Maleimide) and supplemented with DAPI (final dilution: 1:40; Invitrogen). The 2X antibody and DAPI mixture was combined with an equal volume of the residual PBS in each well and incubated with the samples for 4 h under gentle agitation in the dark. Details for each secondary antibody and Fab labeling kit are listed in Supplementary Tables 1 and 2.

5. Optical Clearing by RI Matching: Excess secondary antibodies and dyes were removed by extensive washing with PBS. Optical clearing solution for refractive index matching was prepared as advised by Chung lab protocol: (https://docs.abcam.com/pdf/protocols/clarity-protocol.pdf). N-acetyl-cysteine (Sigma-Aldrich, 0.7 M) was added to this buffer prior to imaging to reduce oxidative damage caused by light exposure during imaging. Lastly, DAPI was added to the imaging buffer in the same concentration used for staining, to prevent its gradual diffusion out of the sample during long imaging time. Embryos were kept in the imaging buffer for at least 1 h prior to imaging to ensure buffer equilibration.

6. Imaging Imaging was performed using a Yokogawa CV8000 spinning disk (CSU-W1) inverted confocal microscope equipped with a 50 μm pinhole disk and a Hamamatsu ORCA Flash 4.0 camera. A 20x/1.0 NA water immersion objective was utilized to capture high-resolution images, with each field of view containing a single embryo. Embryos were detected and selected using a custom Python script. A z-stack of 271 μm was acquired with 1 μm spacing between optical sections. Laser power and exposure time were adjusted individually for each stain to optimize signal quality. Imaging was conducted overnight.

7. RI Matching Buffer Washout: The imaging buffer was washed out thoroughly using PBS after each cycle of imaging.

### Re-staining controls

As a technical control, markers from the first three staining cycles were re-stained in cycle four, each using three replicate wells. Signal correlations between the original stains and their re-stainings were assessed at the mean nuclear level, and at the single-pixel scale using full-resolution or two times down-sampled images (Fig. 1f, g).

### Live imaging and nuclear morphology dynamics

To monitor changes in nuclear size, 0.5 nl of injection mix containing 0.2 µg of 10 kDa Dextran Alexa Fluor (Invitrogen, D1868)^63^ to label the injection site, and 0.8 ng of Histone H1-Alexa Fluor 488 conjugate (Thermo Fisher Scientific, H13188)^35^ to label nuclei, was injected into embryos at the one-cell stage. Live imaging of embryos during the 10^th^ division cycle was performed on an inverted VisiScope spinning-disk microscope equipped with a Nikon 20 × 0.95 NA objective. During imaging, the temperature was maintained at 28.5 ± 1 °C using a stage-heating device. Z-stacks spanning 250 µm were acquired at 1 µm intervals with a temporal resolution of less than 2 min. Using this dataset, 24 nuclei from two embryos were tracked manually using Mastodon^64^. Nuclei were segmented using the Cellpose nuclei model (v2.1.0). To compare these measurements with the fixed multiplexed dataset, the effective nuclear radius in each dataset was calculated as the cubic root of nuclear volume and normalized to the median value over time. The corresponding time and pseudotime axes were also normalized for direct comparison.

### Perturbation experiments

To demonstrate the sensitivity of our method to changes in protein activity, embryos were treated with the RNA polymerase II inhibitor triptolide (Sigma, T3652). For this, embryos were incubated in 14.5 μM triptolide from the one-cell stage until the 10^th^ division cycle. Control embryos were treated with DMSO from the one-cell stage. To investigate the role of the cell cycle in transcriptional activity, dechorionated embryos were treated with a cocktail of 60 mM hydroxyurea (Sigma, H8627) and 300 μM aphidicolin (Sigma, A0781) (HUA) from the 128- to 512-cell stages and maintained in this solution until the 10^th^ division cycle. Control embryos for this condition were treated with DMSO from the 128-cell stage. Immunofluorescence was then performed according to the protocol described above to label Pol II-S2p, PCNA, and β-catenin.

### HDF5 format

An important step in standardizing image analysis workflows is the on-disk specification of data. To streamline this process, we developed a method for storing image data using a custom container based on HDF5. This format is designed to store image pyramids (multiple down-sampled resolutions of raster data) and label images. Here, each microscopy site is written to a HDF5 file, containing groups for each channel and its corresponding resolution levels. Metadata is stored as dataset-level attributes. In the past years, OME-Zarr has emerged as a standard format across the open-source bioimage community^65^ and is thus recommended for new projects.

### Image registration of acquisition cycles

During each cycle of immunofluorescence imaging, sample shrinkage and slight displacements occur due to exposure to elution and imaging buffers and variations in microscope stage initializations, respectively. This necessitates an unsupervised approach to register all acquisitions within a multiplexing experiment to a reference acquisition cycle. To address this, we stained the samples with DAPI in each cycle to label DNA, which serves as a reference channel for registration across cycles. We then utilized the elastix library (itk-elastix v0.13.0)^21,22^, which offers a comprehensive suite of tools for developing custom image registration workflows. Due to embryo shape deformations and stage shifts across cycles, we applied three sequential registration steps, each introducing more degrees of freedom for alignment: starting with rigid (translations and rotations), progressing to affine (rigid, scale, and shearing), and finally applying b-spline (elastic, non-linear transformation) registrations (Supplementary Fig. 1a). The second acquisition cycle was selected as the reference cycle for registration, as it contained the cell segmentation marker (β-catenin). Although these transformations significantly improved the alignment of embryos at single-cell resolution across cycles (Supplementary Fig. 1), some nuclei, particularly those at the borders of the imaged volume, remained misaligned. To assess alignment performance quantitatively, we measured Pearson’s correlation coefficient of nuclear DAPI between cycles. This coefficient was above 0.9 for most cells, and cells with a correlation below this threshold were excluded from downstream analysis (Supplementary Fig. 1b).

### Object segmentation

Embryos were segmented using β-catenin and DAPI staining and by training a pixel classifier in Ilastik(v1.4.0b27)^23^. Nuclei segmentation was performed using the pre-trained Cellpose nuclei model with the expected nuclei diameter set to 25 pixels^24^ (=16.25 µm). To ensure consistent nuclei segmentation across the embryo, the DAPI signal was normalized along the z-axis by applying an exponential rescaling correction with a factor of −0.0045, which improved signal intensity at higher z-planes. This correction was applied solely to enhance segmentation performance; raw DAPI intensities were left unmodified for downstream analyses. For cell segmentation, we implemented a solidity-constrained seeded watershed approach, with nuclei as seeds and embryo mask as foreground, using the scikit-image implementation (v0.19.3)^25^. Membrane labels were generated by selecting the single-pixel outline of the cell labels, and cytoplasm segmentation was obtained by subtracting both the nuclear and membrane labels from the cell masks. Finally, for subnuclear detection of active transcription sites, a second pixel classifier was trained using Pol-II-S2p staining in Ilastik^23^. Segmentation steps were performed on two times down-sampled images, as this resulted in a more isotropic x, y, z voxel spacing compared to the full-resolution images, thereby improving segmentation performance.

### Feature aggregation and extraction

By aggregating features across multiple levels, from individual voxel measurements to objects and hierarchical interactions, we gain a comprehensive understanding of complex biological systems. This approach allows us to study not only individual cells but also their broader tissue context, providing powerful insights into spatially resolved biological data. The structured organization of features into aggregating tables ensures efficient querying, aggregation, and integration into machine learning workflows, making this process scalable for large datasets. This approach is broken down into the following layers:

1. Voxel-level features: Each voxel carries information about its location, extent, fluorescence intensity, and its place in the hierarchical object structure (e.g., nucleus, cell, embryo). These voxel properties serve as the foundation for raster features, such as volume, intensity, and shape (e.g., roundness).

2. Raster features form the most fundamental class of features and are the basis for all downstream measurements. These features include both label-based (nucleus, cell, etc) and intensity-based measurements:

A. Label features: These are computed from label images and include label ID, bounding box, centroid, and morphological properties such as roundness, volume, and Feret diameter^26^.
B. Intensity features: These features describe the intensity distributions within label regions using descriptive statistics (mean, median, kurtosis, etc.)^26^.
C. Colocalization features: These features measure the relationship between staining channels within label regions, quantifying the correlation or mutual information between different fluorescence markers and are useful for studying protein interactions and validating registration quality.
D. Distance features: These features calculate distances between label and reference objects (e.g., from a nucleus to its parent cell). They include centroid distances, maximum, minimum, and median distances based on Euclidean distance transforms.

3. Population features focus on spatial interactions between objects represented as a graph, where nodes are objects and edges depict spatial or hierarchical relationships. We construct four distinct types of graphs:

A. Radius neighborhood: Objects within a given radius are considered neighbors.
B. *K*-nearest neighbors (KNN): Each object is connected to its k closest neighbors, regardless of their distance, ensuring a fixed degree for all nodes.
C. Delaunay triangulation: Neighbors are defined by applying Delaunay triangulation on objects, partitioning the convex hull into tetrahedra.
D. Touch neighborhoods: Adjacency is defined by shared surface area between objects.

4. Hierarchy feature aggregations take place across different levels of the object hierarchy. For instance, by aggregating cells to their parent embryo, the average cell cycle phase or the number of cells in an embryo can be measured. This level provides a global view of embryos/tissues.

5. Feature embeddings include combining multiple feature columns into higher-level properties. These embeddings offer compact representations of complex data and can be either categorical or continuous.

### Intensity bias corrections

To estimate flatfield corrections, the following fluorescent dyes were diluted in DMSO, mixed and added to a Greiner 96-well plate: 7- diethylamino 4-methyl coumarin (Sigma-Aldrich, 0.5 mg/ml), Fluorescein (Sigma-Aldrich, 1 mg/ml), Rose Bengal (Sigma-Aldrich, 1 mg/ml) and acid blue 9 (TCI Chemicals, 10 mg/ml). Multiple imaging stacks were acquired for each wavelength, following the same acquisition protocol as described earlier. Flat fields for each channel were then calculated using the BaSiC Fiji plugin^28^. Dark fields were recorded by averaging 100 images with the laser turned off. Dark and flat field corrections were applied irreversibly during the conversion from raw TIFF images to HDF5 format. The corrected images were clipped at 0 to prevent integer overflow before being cast to uint16. Acquisition time (t-decay) and z intensity (z-decay) bias models were estimated based on the mean intensity features extracted after nuclei segmentation. Both t-decay and z-decay models were estimated separately for each stain, by fitting a single or double exponential curve to the data. Bias corrections were applied to the imaged volumes on the fly prior to final feature extraction. The background signal was estimated by measuring the mean signal intensity in the cytoplasm. This was then subtracted from the mean nuclear signal to obtain mean background-subtracted values. The sum nuclear intensity was subsequently calculated by multiplying the background-subtracted nuclear mean by the nuclear volume.

### Napari feature classifier

We developed a napari feature classifier plugin for annotation and interactive classification of labeled objects: (https://napari-hub.org/plugins/napari-feature-classifier.html). The plugin supports loading measurements and attaching them to a label image and its corresponding multi-channel images in NAPARI. Users define classes and interactively assign training samples, after which a random forest classifier is trained within the interface. Predicted object classes are displayed in real time, enabling iterative refinement of the training set by adding examples in regions where classification performance is sub-optimal. We used this plugin to classify segmented nuclei as debris, enveloping layer (EVL), yolk syncytial layer (YSL), or deep cell labels.

### Cell cycle phase (CCP) inference

The following steps were involved in generating the CCP embedding and hyperparameter tuning:

1. Feature pre-processing:

A. Outlier removal: Removing label objects with fluorescence artifacts in the image channels or mis-segmented nuclei.
B. Stratification: Stratifying the data into division cycle bins based on estimated embryo age, defined as log_2_ (nuclei count). Embryos from cycles 7 to 9 were pooled due to fewer cells at these stages.
C. Sub-sampling: Ensuring an equal number of cells per stratum.

2. Pre-embedding:

A. Feature selection: From the complete feature panel, we selected multiple nuclear morphology features (nuclear volume, roundness and flatness and nucleus/cytoplasmic volume ratio) as well as intensity features of cell cycle markers DAPI sum (DNA content) and mean PCNA (ccp-features in Supplementary Table 3). pH3 was excluded from the features in the final model due to background accumulation.
B. Feature scaling^66^:
  - Standard scaler: Rescaling to 0 mean and 1 standard deviation.
  - Robust scaler: Rescaling to 0 median and 1 quantile range.
  - Power transformer: A monotonic non-linear transformation toward a Gaussian distribution with 0 mean and 1 standard deviation using the ‘Yeo-Johnson’ method.
C. Pre-embedding:
  - An embedding method was selected from scikit-learn compatible dimensionality reduction algorithms (e.g., PCA^67^, UMAP^34^, or Palantir^68^).
  - Embedding dimensionality was specified, with 2-4 dimensions evaluated.
  - Stratification was optional; see “Cell Cycle Phase Scoring Pipeline and Hyperparameter Tuning”.

3. CCP inference:

A. Fit a principal circle to the pre-embedded nuclei of each stratum^69^.
B. CCP embedding: Compute this by fitting a smooth b-spline through the principal circle nodes.
C. CCP inference: Approximate the CCP for each nucleus using:
  - Distance-based inference, where CCP is defined as the position of the closest point on the circle using Euclidean distance.
  - Angle-based inference (only for 2D pre-embedding), where CCP is defined as the angle to the center of mass of the loop.

D. Alignment:
  - Detect peaks and infer their identity (S to M phases) and order using cell cycle markers.
  - Use peak values in the 2^nd^ derivative of mean PCNA intensity to identify the start and end of the S phase.
E. Normalization:
  - To achieve a uniform density distribution for each stratum, we applied the QuantileTransformer from Scikit-learn.

### Cell cycle phase scoring pipeline and hyperparameter tuning

To find the optimal CCP inference, we included as many hyperparameters as possible in the above-mentioned pipeline and evaluated these models by manually classifying 634 cells across different time points into 6 cell cycle classes (anaphase, telophase, early S phase, S phase, prophase, and metaphase of mitosis, as shown in Fig. 3b, b’). This manual cell cycle annotation was performed using DAPI, PCNA and pH3 channels, nuclear morphologies and embryonic context (e.g., mitotic cells in the vicinity) as visual metrics. We assessed the rank ordering of the models by partitioning the model-inferred position of the annotated cells within the embedding into six classes and counting the number of pairwise permutations required to achieve the ground-truth annotated classes. A challenge arose due to the circular nature of the embedding, where the numerical values of 0 and 2π correspond to the same point, meaning metaphase and anaphase are adjacent. To account for this, instead of the usual 5 cuts to partition a one-dimensional feature into six regions, we required six cuts for this circular feature. Each model was evaluated for all strata (cycles 7–9, 10, 11 and 12), and its worst score across these cycles was recorded to ensure the selection of a model that performs reasonably well across all embryonic stages. Each run involved defining a parameter grid and evaluating the resulting models based on the scoring procedure. The key trends that guided the selection of the final model were:

1. **Sub-sampling** was the most significant contributor to model performance, particularly at later cycles that are heavily skewed toward interphase. Such class imbalance hinders correct CCP inference across all phases.
2. **Non-linear feature scaling** outperformed both robust and standard scaling approaches.
3. **Non-linear pre-embeddings** such as UMAP and Palantir outperformed linear ones, e.g., PCA.
4. **Pre-embedding stratification** per division cycle slightly outperformed a single pre-embedding.
5. **Separate principal circle fitting** outperformed a single fit for all strata.

Since subsampling emerged as a major determinant of model performance, we repeated the procedure by using the initially selected CCP model to guide a new round of data subsampling. The model was then retrained on this resampled dataset, which yielded qualitatively improved results.

We note that although the global structure of the CCP trajectory resembles cell cycle progression, as indicated by the gradual doubling of total DNA content during interphase (DAPI sum, Fig. 4a), the local positioning of cells within the CCP may contain errors. For example, some mitotic nuclei resembling the metaphase-to-anaphase transition are positioned at prophase according to CCP inference, likely due to their morphological similarity to prophase nuclei. Such errors should not influence downstream prediction analyses, as mitotic cells were excluded.

### Embryo staging

Since embryos of different timepoints were mixed in the same well to minimize technical variability, they were first re-staged based on nuclei counts during data analysis steps (Fig. 2i). However, this method provides only cycle-level resolution, and missing nuclei can be misinterpreted as differences in developmental age. To overcome this limitation, following nuclei count-based staging, embryos were further ordered according to their circular mean CCP, thereby refining developmental staging (CCP-adjusted staging, Supplementary Fig. 4b).

### Cell cycle phase circular statistics

CCP is a circular variable ranging from 0 to 2π, with the endpoints representing the same phase of the cell cycle. To account for this periodicity, we used Scipy’s circular statistics^70^: the circular mean *Θ* (scipy.stats.circmean) to compute the average CCP per embryo and circular synchrony *R* as *R* = 1-scipy.stats.circvar, measuring the reduction in CCP variance across cells within each embryo (Supplementary Fig. 4b and 3e). To estimate local CCP synchrony (Fig. 3f), “*R*” was calculated by aggregating cells in radius-defined neighborhoods ranging from 0 to 500 μm.

### Modeling global cell cycle synchrony

We modeled R as a function of CCP-adjusted staging (*s*) using a four-parameter Hill equation:

1 $$R={R}_{max }+\frac{{R}_{min }-{R}_{max }}{1+{({s}_{{inflection}}/s)}^{n}}$$

where *R*_max_ and *R*_min_ are the asymptotic maximum and minimum synchrony, *s*_inflection_ is the stage at half-maximal synchrony and *n* is the Hill coefficient. Parameters were estimated by non-linear least squares using SciPy’s scipy.optimize.curve_fit. Pointwise confidence intervals for the mean Hill curve were obtained via a residual bootstrap, where, after the initial fit, the residuals (observed *R* minus fitted *R*) were resampled and added to the fitted values to create synthetic responses (200 datasets). The Hill model was refit for each bootstrap sample, and the resulting mean and 95% confidence intervals were reported.

### Cell cycle phase gradient estimation and pseudowave reconstruction

CCP gradients were computed using a finite difference method designed for irregularly spaced data^71^. The method was adapted to operate on 3D point clouds and modified to incorporate circular differences, enabling application to CCP. The resulting gradient field was integrated with PyVista to generate streamlines for visualizing mitotic pseudowaves (Fig. 3g). We note that the gradient-based method used to visualize mitotic pseudowaves is intrinsically less reliable near tissue boundaries and at early stages with few nuclei, because local gradients are then estimated from one-sided and often sparse neighbor configurations^71^.

### Modeling of transcriptional activity within division cycles

We modeled transcriptional activity during S phase using natural log-transformed mean nuclear Pol-II-S2p intensity, as a proxy for transcriptional output^3,19^ by performing multilinear regression. The beginning and end of the S phase were defined using peak values in the 2^nd^ derivative of mean PCNA intensity. Regression models were formulated in a Bayesian framework (using PyMC^72^ and Bambi^73^) with posterior inference obtained by Markov Chain Monte Carlo sampling, and model diagnostics performed using ArviZ library^74^. The response variable, y, was assumed to be normally distributed:

2 $${\rm{y}}\, \sim {\rm{Normal}}\,(\mu,\sigma )$$

with expected value μ and standard deviation *σ*. We tested a range of model specifications for their expected values. Linear models included predictors (*X*_linear_) such as CCP and ZGA factors (zga; including Pou5f3, Nanog, H3K27ac, H2B). Additionally, we included features used for CCP inference as linear predictors in our modeling approach (ccp-feats: PCNA and DAPI intensities (ccp-intensity), nuclear volume and shape descriptors (morphology), see Supplementary Table 3), as they may capture additional information beyond that represented by CCP. These linear models are depicted as:

3 $$\mu={\beta }_{0}+\beta {X}_{{linear}}$$

with ${\beta }_{{\boldsymbol{0}}}$ and β assuming to follow normal distributions.

As the relationship between CCP and Pol-II-S2p was found to be non-linear (Fig. 4b), we additionally implemented asymptotic, power (Eqs. 4 and 5) and b-spline (3 knots without intercept) regression models of ${X}_{{CCP}}$:

4 $$\mu \,=\,a-(a-b){exp }^{({-{cX}}_{{CCP}})}$$

5 $$\mu \,=\,a{{X}_{{CCP}}}^{b}$$

Finally, these non-linear CCP formulations were also combined with linear predictors to capture all features that vary non/linearly in explaining transcriptional activity, as shown for asymptotic and power models below:

6 $$\mu \,=\,a-(a-b){exp }^{({-{cX}}_{{CCP}})}+\,\beta {X}_{{linear}}$$

7 $$\mu \,=\,a{{X}_{{CCP}}}^{b}+\,\beta {X}_{{linear}}$$

with coefficients a, b and c assuming to follow normal distributions. For the custom PyMC asymptotic model, priors were: *σ* ~ HalfCauchy(2), asymptote ~ Normal(5,2), intercept ~ Normal(0,1), rate ~ Gamma(10,2.5), and linear coefficients ~ Normal(0,1). For the custom PyMC power model, priors were: *σ* ~ HalfCauchy(2), *a* ~ Normal(0,2), *b* ~ Normal(0,3), and linear coefficients ~ Normal(0,1). Bambi models used default priors. Custom PyMC models were sampled with 3000 posterior draws per chain.

Model names indicate included feature sets. For example, the model *zga_ccp_ccp-power* combined linear predictors (ZGA factors and CCP) with a non-linear power regression on CCP. For the asymptotic and power regression models, we tried additional models by clipping/censorting the data at 100 gray values, as detection accuracy drops at these low signal-to-noise ratios (termed ccp-cens-asympt, ccp-cens-power). Intensity features used for prediction were first natural log-transformed and, together with morphology features, z-scored to enable direct comparison across features. Models were trained on up to 500,000 cells per division cycle (cycles 8-12). Predictive performance was assessed using explained variance (*R*²) and expected log pointwise predictive density (ELPD). The best performing model, zga_ccp-feats_ccp-asympt, was trained for each division cycle separately and evaluated on exemplary embryos excluded from training steps in that cycle (Fig. 4g, g’).

### Modeling of transcriptional activity across division cycles

To model transcriptional activity across division cycles, we divided S phase into six equally sized strata during which the S phase marker PCNA remained unchanged. Within each stratum, with CCP held constant, we trained multilinear regression models to predict transcriptional output (natural log-transformed mean nuclear Pol-II-S2p intensity) from z-scored and natural log-transformed ZGA factor levels, enabling direct comparison of regression coefficients. Model performance was evaluated using 5-fold cross-validation, and predictive accuracy was summarized by *R*² calculated from the combined S phase data. Regression coefficients for each stain were estimated separately within each stratum, and their distributions are shown in Fig. 4f^’^. Permutation analysis was performed by shuffling the values of each marker and measuring the resulting reduction in *R*² in Supplementary Fig. 6d.

### Statistics and reproducibility

For the Bayesian analyses, details on prior selection and Markov chain Monte Carlo settings are provided in the Methods. Statistical analyses were performed in Python using SciPy, and Bayesian regression modeling was carried out using PyMC, Bambi, and ArviZ. For the multiplexing dataset, 212 embryos were collected between 2 and 4 h post-fertilization. Given that the earlier division cycles last approximately 15–20 min, this sampling provided sufficient temporal resolution to capture the biological processes studied here. Outliers, as well as mis-segmented or misaligned nuclei and cells, were excluded from downstream analyses. All experiments and image analyses were done through streamlined pipelines, in which all conditions were normalized and analyzed similarly.

### Reporting summary

Further information on research design is available in the Nature Portfolio Reporting Summary linked to this article.

## Supplementary information

Supplementary Information

Reporting Summary

Transparent Peer Review file

## Source data

Source Data

## Data Availability

The multiplexed data generated in this study have been deposited in the BioImage Archive database under the accession number S-BIAD3228. Any additional data can be provided by the corresponding author of this study. Source data are provided with this paper.

## Code availability

All code used for the image analysis pipelines is available via GitHub at (https://github.com/pelkmanslab/abbott/tree/v0.3.9.1), (https://doi.org/10.5281/zenodo.22012790, https://github.com/pelkmanslab/abbott-features/tree/v0.1.5.2),(https://doi.org/10.5281/zenodo.22012817), (https://github.com/pelkmanslab/abbott-segmentation-tasks/tree/v0.4.2), (https://doi.org/10.5281/zenodo.22012831) as provided here^75^ and (https://github.com/MaksHess/manuscript-multiplexed-embryo-profiling).

## References

[1] Vastenhouw, N. L., Cao, W. X. & Lipshitz, H. D. The maternal-to-zygotic transition revisited. *Development* **146**, dev161471 (2019). 10.1242/dev.161471 31189646

[2] Heyn, P. et al. The earliest transcribed zygotic genes are short, newly evolved, and different across species. *Cell Rep.* **6**, 285–292 (2014). 10.1016/j.celrep.2013.12.030 24440719

[3] Hadzhiev, Y. et al. A cell cycle-coordinated Polymerase II transcription compartment encompasses gene expression before global genome activation. *Nat. Commun.* **10**, 691 (2019). 10.1038/s41467-019-08487-5 PMC6370886 30741925

[4] Kimelman, D., Kirschner, M. & Scherson, T. The events of the midblastula transition in Xenopus are regulated by changes in the cell cycle. *Cell* **48**, 399–407 (1987). 10.1016/0092-8674(87)90191-7 3802197

[5] Collart, C., Allen, G. E., Bradshaw, C. R., Smith, J. C. & Zegerman, P. Titration of four replication factors is essential for the Xenopus laevis midblastula transition. *Science* **341**, 893–896 (2013). 10.1126/science.1241530 PMC3898016 23907533

[6] Joseph, S. R. et al. Competition between histone and transcription factor binding regulates the onset of transcription in zebrafish embryos. *eLife* **6**, e23326 (2017). 10.7554/eLife.23326 PMC5451213 28425915

[7] Chan, S. H. et al. Brd4 and P300 confer transcriptional competency during zygotic genome activation. *Dev. Cell* **49**, 867–881.e8 (2019). 10.1016/j.devcel.2019.05.037 PMC7201981 31211993

[8] Lee, M. T. et al. Nanog, Pou5f1 and SoxB1 activate zygotic gene expression during the maternal-to-zygotic transition. *Nature* **503**, 360–364 (2013). 10.1038/nature12632 PMC3925760 24056933

[9] Pálfy, M., Schulze, G., Valen, E. & Vastenhouw, N. L. Chromatin accessibility established by Pou5f3, Sox19b and Nanog primes genes for activity during zebrafish genome activation. *PLoS Genet*. **16**, e1008546 (2020). 10.1371/journal.pgen.1008546 PMC6986763 31940339

[10] Wu, E. & Vastenhouw, N. L. From mother to embryo: A molecular perspective on zygotic genome activation. *Curr. Top.Dev. Biol*. **140**, 209–254 (Elsevier, 2020). 10.1016/bs.ctdb.2020.02.002 32591075

[11] Kuznetsova, K. et al. Nanog organizes transcription bodies. *Curr. Biol.* **33**, 164–173.e5 (2023). 10.1016/j.cub.2022.11.015 36476751

[12] Pelkmans, L. Using cell-to-cell variability—a new era in molecular biology. *Science* **336**, 425–426 (2012). 10.1126/science.1222161 22539709

[13] Kramer, B. A., Sarabia del Castillo, J. & Pelkmans, L. Multimodal perception links cellular state to decision making in single cells. *Science* eabf4062 10.1126/science.abf4062 (2022). 35857483

[14] Battich, N., Stoeger, T. & Pelkmans, L. Control of transcript variability in single mammalian cells. *Cell* **163**, 1596–1610 (2015). 10.1016/j.cell.2015.11.018 26687353

[15] Gut, G., Herrmann, M. D. & Pelkmans, L. Multiplexed protein maps link subcellular organization to cellular states. *Science* **361**, eaar7042 (2018). 10.1126/science.aar7042 30072512

[16] Wahle, P. et al. Multimodal spatiotemporal phenotyping of human retinal organoid development. *Nat. Biotechnol*10.1038/s41587-023-01747-2 (2023). PMC10713453 37156914

[17] Mishra, N., Li, Y. I., Hannezo, E. & Heisenberg, C.-P. Geometry-driven asymmetric cell divisions pattern cell cycles and zygotic genome activation in the zebrafish embryo. *Nat. Phys.* **22**, 139–150 (2026).

[18] Zhang, M., Kothari, P., Mullins, M. & Lampson, M. A. Regulation of zygotic genome activation and DNA damage checkpoint acquisition at the mid-blastula transition. *Cell Cycle* **13**, 3828–3838 (2014). 10.4161/15384101.2014.967066 PMC4614548 25558827

[19] Ugolini, M. et al. Transcription bodies regulate gene expression by sequestering CDK9. *Nat. Cell Biol.* **26**, 604–612 (2024). 10.1038/s41556-024-01389-9 PMC11021188 38589534

[20] Schindelin, J. et al. Fiji: an open-source platform for biological-image analysis. *Nat. Methods* **9**, 676–682 (2012). 10.1038/nmeth.2019 PMC3855844 22743772

[21] Ntatsis, K. et al. itk-elastix: Medical image registration in Python. in 101–105 10.25080/gerudo-f2bc6f59-00d (Austin, Texas, 2023).

[22] Marstal, K., Berendsen, F., Staring, M. & Klein, S. *SimpleElastix: A User-Friendly, Multi-lingual Library for Medical Image Registration*. in *2016**IEEE Conference on Computer Vision and Pattern Recognition Workshops (CVPRW**)* 574–582 10.1109/CVPRW.2016.78 (IEEE, 2016).

[23] Berg, S. et al. ilastik: interactive machine learning for (bio)image analysis. *Nat. Methods* **16**, 1226–1232 (2019). 10.1038/s41592-019-0582-9 31570887

[24] Stringer, C., Wang, T., Michaelos, M. & Pachitariu, M. Cellpose: a generalist algorithm for cellular segmentation. *Nat. Methods* **18**, 100–106 (2021). 10.1038/s41592-020-01018-x 33318659

[25] Van Der Walt, S. et al. scikit-image: image processing in Python. *PeerJ* **2**, e453 (2014). 10.7717/peerj.453 PMC4081273 25024921

[26] McCormick, M., Liu, X., Jomier, J., Marion, C. & Ibanez, L. ITK: enabling reproducible research and open science. *Front. Neuroinform*. **8**, 13 (2014). 10.3389/fninf.2014.00013 PMC3929840 24600387

[27] Sofroniew, N. et al. Napari: a multi-dimensional image viewer for Python. *Zenodo* 10.5281/ZENODO.14719463 (2025).

[28] Peng, T. et al. A BaSiC tool for background and shading correction of optical microscopy images. *Nat. Commun.* **8**, 14836 (2017). 10.1038/ncomms14836 PMC5472168 28594001

[29] Keller, P. J., Schmidt, A. D., Wittbrodt, J. & Stelzer, E. H. K. Reconstruction of zebrafish early embryonic development by scanned light sheet microscopy. *Science* **322**, 1065–1069 (2008). 10.1126/science.1162493 18845710

[30] Kondakova, E. A. A. & Efremov, V. I. I. Morphofunctional transformations of the yolk syncytial layer during zebrafish development. *J. Morphol.* **275**, 206–216 (2014). 10.1002/jmor.20209 24122838

[31] Kwasnieski, J. C., Orr-Weaver, T. L. & Bartel, D. P. Early genome activation in *Drosophila* is extensive with an initial tendency for aborted transcripts and retained introns. *Genome Res*. **29**, 1188–1197 (2019). 10.1101/gr.242164.118 PMC6633261 31235656

[32] Stapel, L. C., Zechner, C. & Vastenhouw, N. L. Uniform gene expression in embryos is achieved by temporal averaging of transcription noise. *Genes Dev.* **31**, 1635–1640 (2017). 10.1101/gad.302935.117 PMC5647934 28903980

[33] Gut, G., Tadmor, M. D., Pe’er, D., Pelkmans, L. & Liberali, P. Trajectories of cell-cycle progression from fixed cell populations. *Nat. Methods* **12**, 951–954 (2015). 10.1038/nmeth.3545 PMC6004611 26301842

[34] McInnes, L., Healy, J. & Melville, J. UMAP: Uniform manifold approximation and projection for dimension reduction. Preprint at 10.48550/arXiv.1802.03426 (2020).

[35] Liu, Z., Woo, S. & Weiner, O. D. Nodal signaling has dual roles in fate specification and directed migration during germ layer segregation. *Development* **145**, dev163535 10.1242/dev.163535 (2018). PMC6141772 30111654

[36] Schulz, K. N. & Harrison, M. M. Mechanisms regulating zygotic genome activation. *Nat. Rev. Genet*. **20**, 221–234 (2019). 10.1038/s41576-018-0087-x PMC6558659 30573849

[37] Sato, Y. et al. Histone H3K27 acetylation precedes active transcription during zebrafish zygotic genome activation as revealed by live-cell analysis. *Development* **146**, dev179127 (2019). 10.1242/dev.179127 PMC6803375 31570370

[38] Liu, J. et al. Dissecting the regulatory logic of specification and differentiation during vertebrate embryogenesis. Preprint at 10.1101/2024.08.27.609971 (2024).

[39] Morita, H. et al. The physical basis of coordinated tissue spreading in zebrafish gastrulation. *Dev. Cell* **40**, 354–366.e4 (2017). 10.1016/j.devcel.2017.01.010 PMC5364273 28216382

[40] Petridou, N. I., Grigolon, S., Salbreux, G., Hannezo, E. & Heisenberg, C.-P. Fluidization-mediated tissue spreading by mitotic cell rounding and non-canonical Wnt signalling. *Nat. Cell Biol.* **21**, 169–178 (2019). 10.1038/s41556-018-0247-4 30559456

[41] Chen, H., Einstein, L. C., Little, S. C. & Good, M. C. Spatiotemporal patterning of zygotic genome activation in a model vertebrate embryo. *Dev. Cell* **49**, 852–866 (2019). 10.1016/j.devcel.2019.05.036 PMC6655562 31211992

[42] McCleland, M. L. & O’Farrell, P. H. RNAi of mitotic cyclins in Drosophila uncouples the nuclear and centrosome cycle. *Curr. Biol.* **18**, 245–254 (2008). 10.1016/j.cub.2008.01.041 PMC2698964 18291653

[43] Fabrèges, D. et al. Temporal variability and cell mechanics control robustness in mammalian embryogenesis. *Science* **386**, eadh1145 (2024). 10.1126/science.adh1145 39388574

[44] Serra, D. et al. Self-organization and symmetry breaking in intestinal organoid development. *Nature* **569**, 66–72 (2019). 10.1038/s41586-019-1146-y PMC6544541 31019299

[45] Priya, R. et al. Tension heterogeneity directs form and fate to pattern the myocardial wall. *Nature* **588**, 130–134 (2020). 10.1038/s41586-020-2946-9 33208950

[46] Gruenheit, N. et al. Cell cycle heterogeneity can generate robust cell type proportioning. *Dev. Cell* **47**, 494–508.e4 (2018). 10.1016/j.devcel.2018.09.023 PMC6251973 30473004

[47] Akieda, Y. et al. Cell competition corrects noisy Wnt morphogen gradients to achieve robust patterning in the zebrafish embryo. *Nat. Commun.* **10**, 4710 (2019). 10.1038/s41467-019-12609-4 PMC6797755 31624259

[48] Aoki, K. et al. Mechano-gradients drive morphogen-noise correction to ensure robust patterning. *Sci. Adv.* **10**, eadp2357 (2024). 10.1126/sciadv.adp2357 PMC11567007 39546611

[49] De Belly, H. et al. Membrane tension gates ERK-mediated regulation of pluripotent cell fate. *Cell Stem. Cell* **28**, 273–284.e6 (2021). 10.1016/j.stem.2020.10.018 PMC7875115 33217323

[50] Rogers, K. W. & Schier, A. F. Morphogen gradients: from generation to interpretation. *Annu. Rev. Cell Dev. Biol.* **27**, 377–407 (2011). 10.1146/annurev-cellbio-092910-154148 21801015

[51] Camacho-Aguilar, E. et al. Combinatorial interpretation of BMP and WNT controls the decision between primitive streak and extraembryonic fates. *Cell Syst*10.1016/j.cels.2024.04.001 (2024). PMC11231731 38692274

[52] Bunne, C. et al. Learning single-cell perturbation responses using neural optimal transport. *Nat. Methods* **20**, 1759–1768 (2023). 10.1038/s41592-023-01969-x PMC10630137 37770709

[53] Wan, Y. et al. Whole-embryo spatial transcriptomics at subcellular resolution from gastrulation to organogenesis. *Science* **391**, eadt3439 (2026). 10.1126/science.adt3439 41818362

[54] Briggs, J. A. et al. The dynamics of gene expression in vertebrate embryogenesis at single-cell resolution. *Science* **360**, eaar5780 (2018). 10.1126/science.aar5780 PMC6038144 29700227

[55] Farrell, J. A. et al. Single-cell reconstruction of developmental trajectories during zebrafish embryogenesis. *Science* **360**, eaar3131 (2018). 10.1126/science.aar3131 PMC6247916 29700225

[56] Kimmel, C. B., Ballard, W. W., Kimmel, S. R., Ullmann, B. & Schilling, T. F. Stages of embryonic development of the zebrafish. *Dev. Dyn.* **203**, 253–310 (1995). 10.1002/aja.1002030302 8589427

[57] Moreno-Mateos, M. A. et al. CRISPR-Cpf1 mediates efficient homology-directed repair and temperature-controlled genome editing. *Nat. Commun.* **8**, 2024 (2017). 10.1038/s41467-017-01836-2 PMC5722943 29222508

[58] Labun, K. et al. CHOPCHOP v3: expanding the CRISPR web toolbox beyond genome editing. *Nucleic Acids Res.* **47**, W171–W174 (2019). 10.1093/nar/gkz365 PMC6602426 31106371

[59] Smutny, M. et al. Friction forces position the neural anlage. *Nat. Cell Biol.* **19**, 306–317 (2017). 10.1038/ncb3492 PMC5635970 28346437

[60] Shamipour, S., Hofmann, L., Steccari, I., Kardos, R. & Heisenberg, C.-P. Yolk granule fusion and microtubule aster formation regulate cortical granule translocation and exocytosis in zebrafish oocytes. *PLoS Biol.* **21**, e3002146 (2023). 10.1371/journal.pbio.3002146 PMC10284390 37289834

[61] Chung, K. & Deisseroth, K. CLARITY for mapping the nervous system. *Nat. Methods* **10**, 508–513 (2013). 10.1038/nmeth.2481 23722210

[62] Susaki, E. A. et al. Versatile whole-organ/body staining and imaging based on electrolyte-gel properties of biological tissues. *Nat. Commun.* **11**, 1982 (2020). 10.1038/s41467-020-15906-5 PMC7184626 32341345

[63] Shamipour, S. et al. Bulk actin dynamics drive phase segregation in zebrafish oocytes. *Cell* **177**, 1463–1479.e18 (2019). 10.1016/j.cell.2019.04.030 31080065

[64] Girstmair, J. et al. Mastodon: the command center for large-scale lineage-tracing microscopy datasets. Preprint at 10.64898/2025.12.10.693416 (2025). 42841415

[65] Moore, J. et al. OME-Zarr: a cloud-optimized bioimaging file format with international community support. *Histochem Cell Biol.* **160**, 223–251 (2023). 10.1007/s00418-023-02209-1 PMC10492740 37428210

[66] Buitinck, L. et al. API design for machine learning software: experiences from the scikit-learn project. Preprint at 10.48550/ARXIV.1309.0238 (2013).

[67] Schwabe, D., Formichetti, S., Junker, J. P., Falcke, M. & Rajewsky, N. The transcriptome dynamics of single cells during the cell cycle. *Mol. Syst. Biol.* **16**, e9946 (2020). 10.15252/msb.20209946 PMC7672610 33205894

[68] Setty, M. et al. Characterization of cell fate probabilities in single-cell data with Palantir. *Nat. Biotechnol.* **37**, 451–460 (2019). 10.1038/s41587-019-0068-4 PMC7549125 30899105

[69] Albergante, L. et al. Robust and scalable learning of complex intrinsic dataset geometry via ElPiGraph. *Entropy* **22**, 296 (2020). 10.3390/e22030296 PMC7516753 33286070

[70] Virtanen, P. et al. SciPy 1.0: fundamental algorithms for scientific computing in Python. *Nat. Methods* **17**, 261–272 (2020). 10.1038/s41592-019-0686-2 PMC7056644 32015543

[71] Meyer, T. H., Eriksson, M. & Maggio, R. C. Gradient estimation from irregularly spaced data sets. *Math. Geol.* **33**, 693–717 (2001).

[72] Abril-Pla, O. et al. PyMC: a modern, and comprehensive probabilistic programming framework in Python. *PeerJ Comput. Sci.* **9**, e1516 (2023). 10.7717/peerj-cs.1516 PMC10495961 37705656

[73] Capretto, T. et al. Bambi: a simple interface for fitting Bayesian linear models in Python. *J. Stat. Soft*. **103**, 1–29 (2022).

[74] Kumar, R., Carroll, C., Hartikainen, A. & Martin, O. ArviZ a unified library for exploratory analysis of Bayesian models in Python. *JOSS* **4**, 1143 (2019).

[75] Hornbachner, R. et al. Shield-4i: a whole-mount multiplexed imaging platform for studying multiscale information flow in 3D multicellular systems. Preprint at 10.64898/2026.07.01.735871 (2026).

[76] Vastenhouw, N. L. et al. Chromatin signature of embryonic pluripotency is established during genome activation. *Nature* **464**, 922–926 (2010). 10.1038/nature08866 PMC2874748 20336069

