# Supplementary material for: Multiplexed embryo profiling links cellular state to zygotic genome activation in single cells
Source: Nat Commun. 2026 Sep 17;17:10929. doi: 10.1038/s41467-026-77784-7 (PMC13680386; doi:10.1038/s41467-026-77784-7)
Supplement: Supplementary file 1 [file 41467_2026_77784_MOESM1_ESM.pdf]

Supplementary Information

Supplementary Figures:

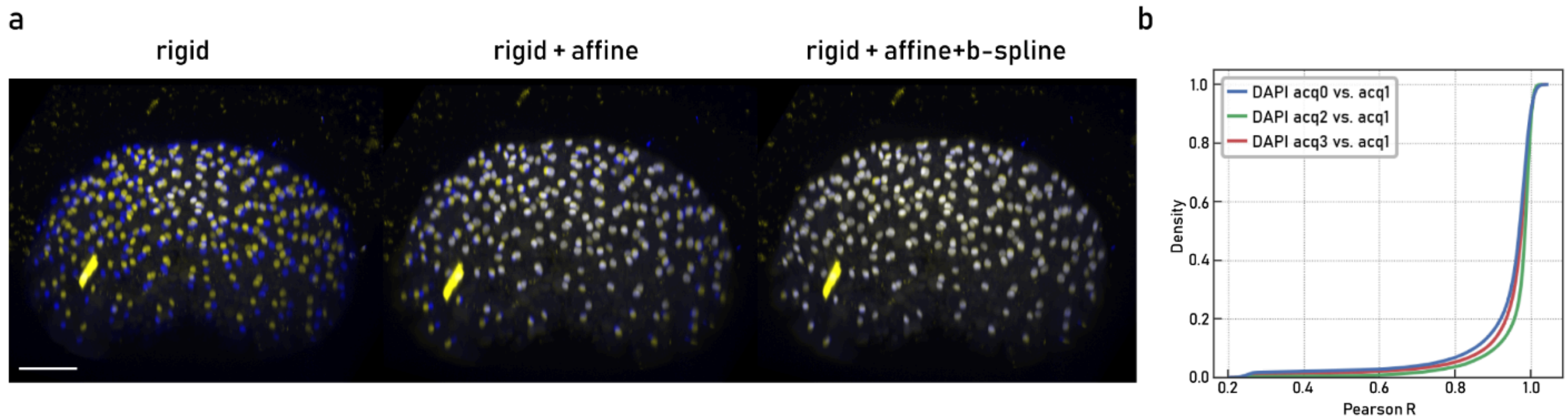

Supplementary Fig. 1: Acquisition cycle registration.

a. DAPI stainings of a representative embryo imaged across two acquisition cycles (blue is the reference cycle and yellow the aligned cycle) after rigid (left), rigid and affine (middle) or rigid, affine and b-spline registration (right) steps.

b. Cumulative distribution of nuclear DAPI correlations from acquisition (acq) cycles 0, 2 or 3 with the reference cycle 1 across all embryos after registration, used as single-cell estimates of registration fidelity. Same data as in Fig. 1. Source data are provided as a Source Data file.

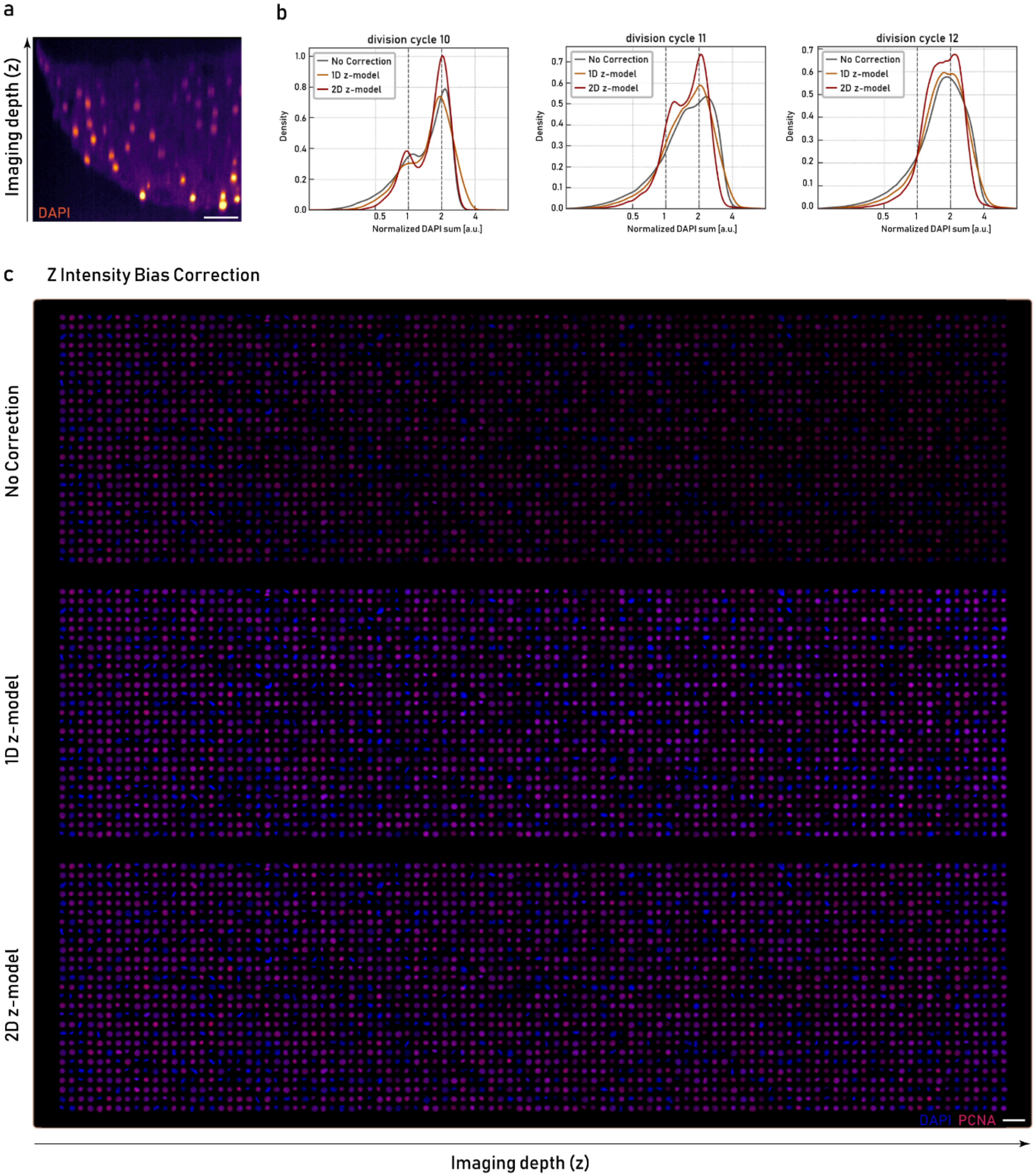

Supplementary Fig. 2: Z intensity bias correction.

a. A representative embryo stained with DAPI showing intensity decay across the imaging depth/z axis (animal pole is at the coverslip). Scale bar 50 µm.

b. The density plot on comparison of 1D and 2D z-decay models with uncorrected data for summed DAPI intensity of embryos in cycles 10 to 12. Data normalized to the first peak.

c. Maximum intensity projections of representative nuclei stained with DAPI (blue) and PCNA (red). Top: uncorrected data, middle: after 1D z-decay correction and bottom: after 2D z-decay correction. Nuclei are sorted by z-coordinate (left

to right corresponds to increasing imaging depth (z)). Scale bar 50 µm. Same data as in Fig. 1. Source data are provided as a Source Data file.

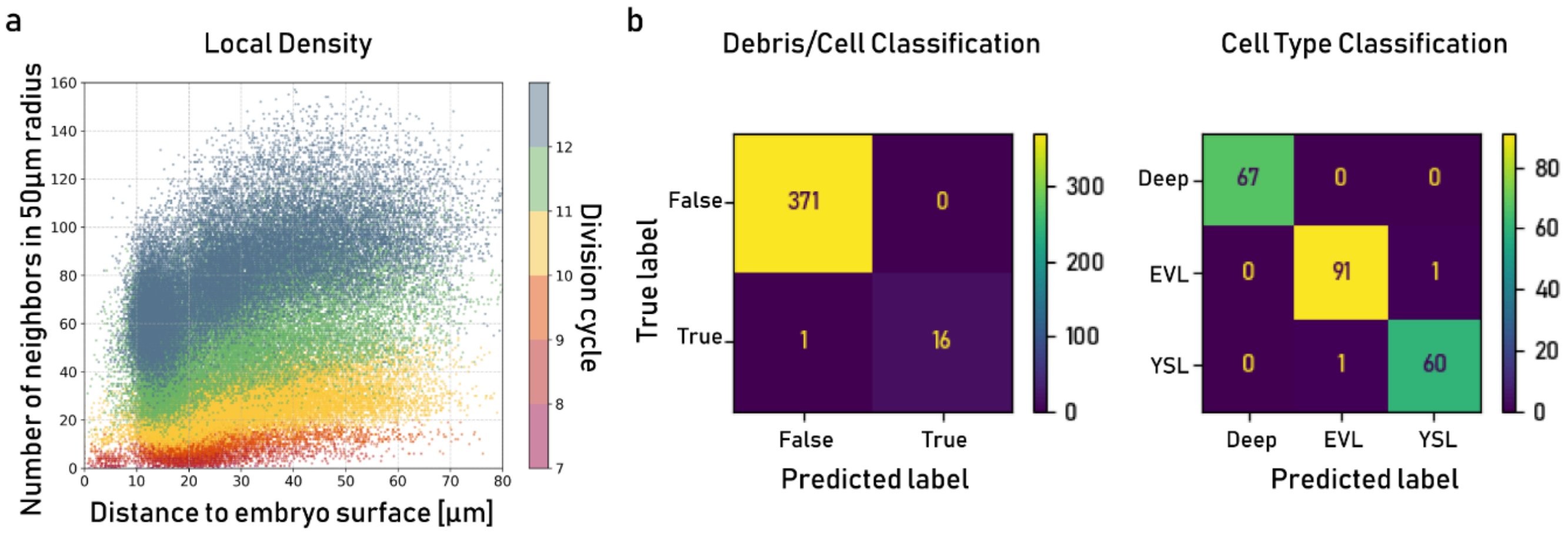

Supplementary Fig. 3: Local density and label object classification.

a. Number of neighbors in a 50 µm radius for each cell plotted against its distance to the embryo surface. Results are color-coded per division cycle. Maximum 30,000 cells for each cycle are shown.

b. Confusion matrices for the two napari feature classifiers, debris vs. cells (left) and deep cells vs. EVL and YSL (right), evaluated on the test set. Same data as in Fig. 1. Source data are provided as a Source Data file.

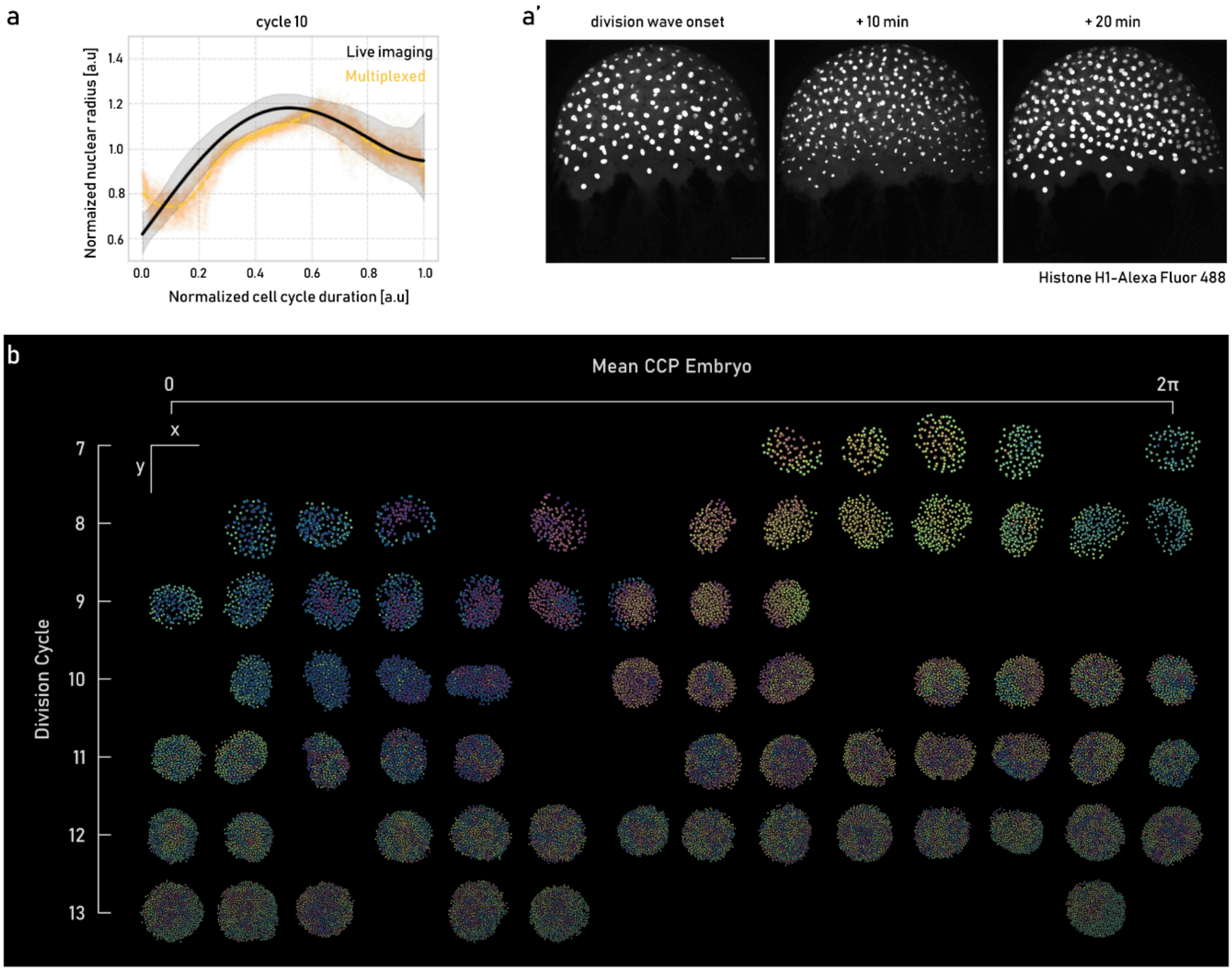

Supplementary Fig. 4: CCP inference validation and refined embryo staging.

a, Comparison of normalized nuclear radius over normalized cell cycle duration between live-imaged embryos and the fixed multiplexed dataset during the 10th division cycle. For the live-imaging dataset, 24 nuclei from two embryos were tracked (N=2 experiments, n=2 embryos). Shaded areas indicate the 90% confidence interval around the median.

a′, Snapshot images of a representative embryo injected with Histone H1-Alexa Fluor 488 conjugate to label and track nuclei, shown at the onset of the division wave and 10 and 20 min later. Scale bar, 100 µm.

b. Top view (along the z-axis) of a subset of embryos across the full developmental time course studied here, with nuclei colored by inferred CCP. Embryos are ordered by division cycle (top to bottom) and CCP circular mean (left to right). Each grid point corresponds to an average CCP bin and a division cycle; empty bins indicate no matching embryo. Scale bars: 550 µm (*x* and *y*). Same data as in Fig. 1. Source data are provided as a Source Data file.

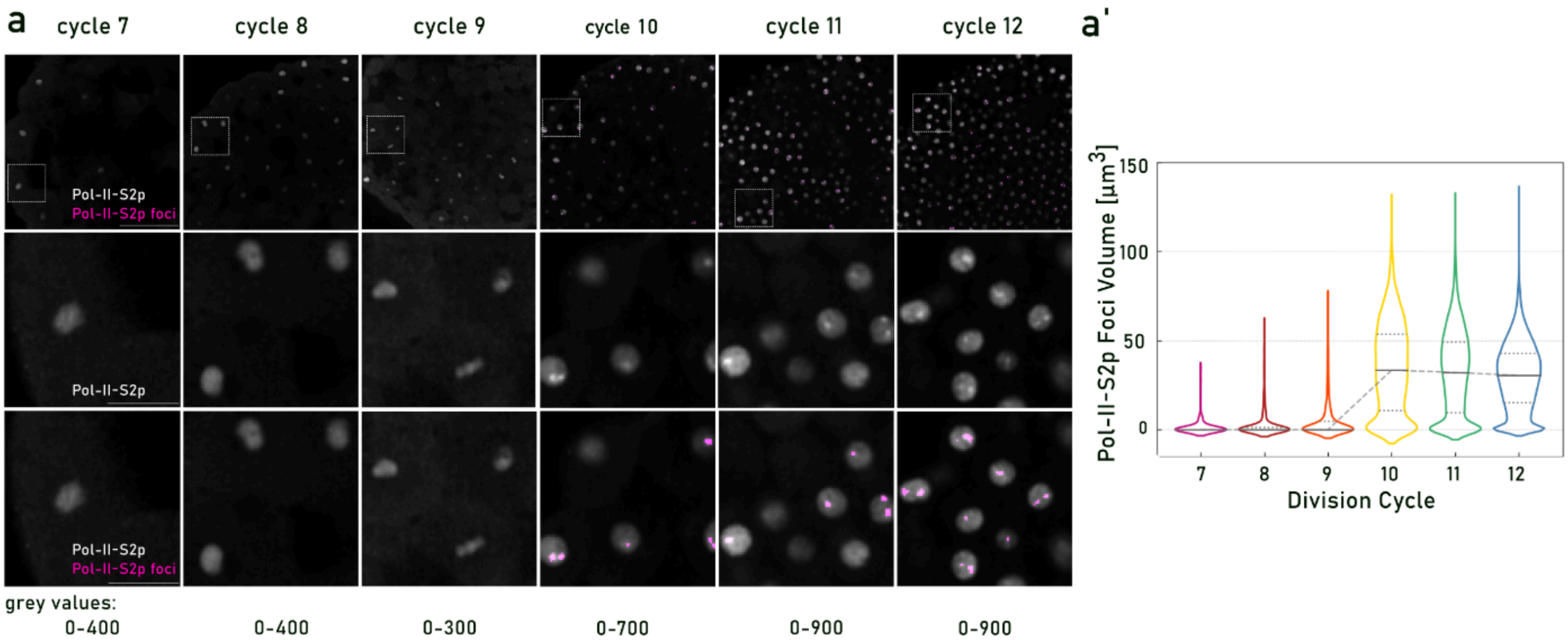

Supplementary Fig. 5: Active transcription sites measurements.

a. 2D sections of representative embryos stained with Pol-II-S2p antibody across division cycles 7 to 12, overlaid with active transcription foci labels detected by Ilastik pixel classifier (top and bottom rows). The last two rows demarcate the zoom-in views of the ROIs shown in top panels. Scale bars in overview and zoom-in views are 100 and 25 µm, respectively.

a'. Active transcription foci volume measurements for all deep-cell nuclei in S phase across division cycles 7 to 12. The dashed line connects the median values for each division cycle. Dotted inner lines: quartiles (Q1 and Q3) for each cycle. Distributions show the full data range. Same data as in Fig. 1. Source data are provided as a Source Data file.

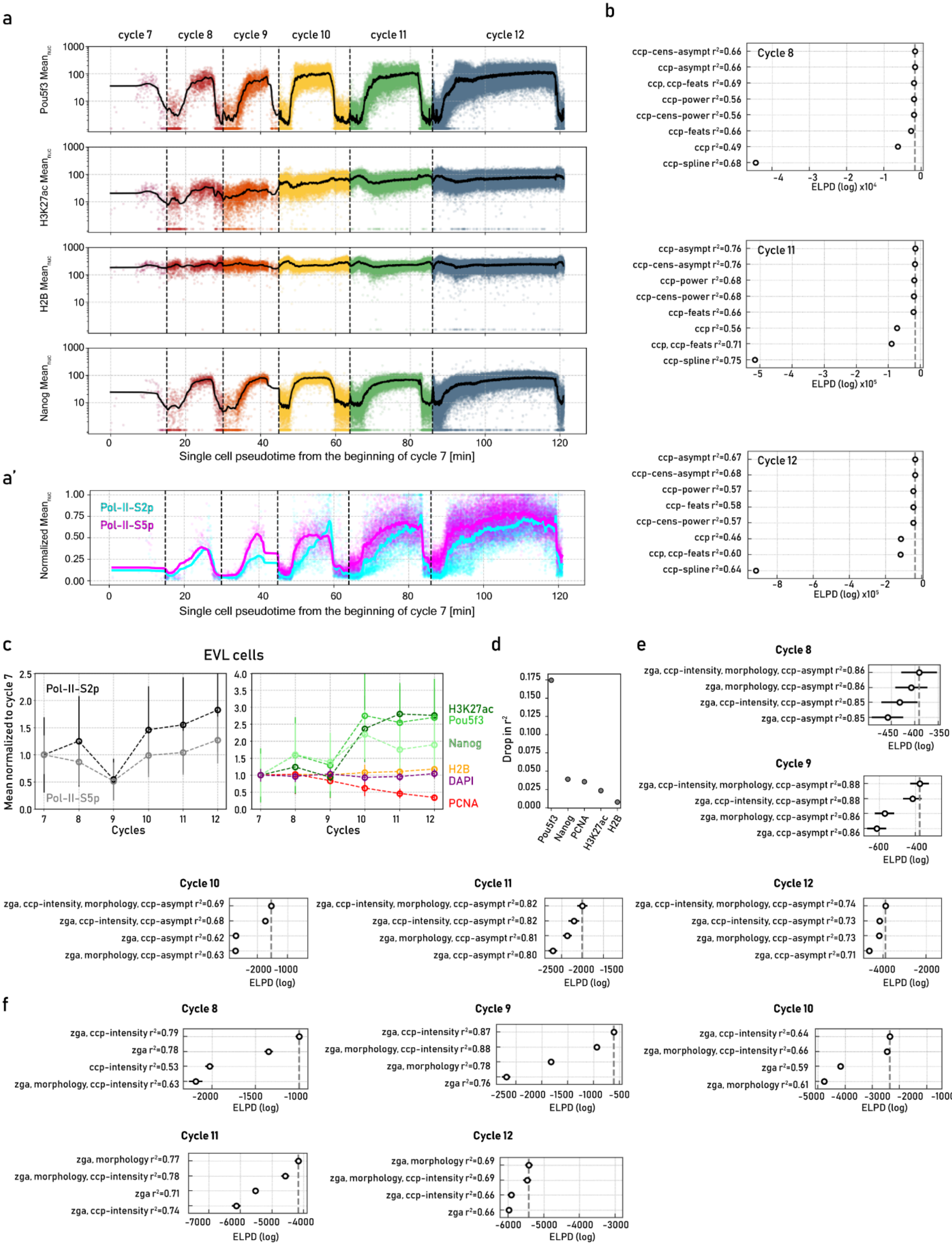
a
cycle 7
cycle 8
cycle 9
cycle 10
cycle 11
cycle 12
Pou5f3 Mean$_{nuc}$
H3K27ac Mean$_{nuc}$
H2B Mean$_{nuc}$
Nanog Mean$_{nuc}$
Single cell pseudotime from the beginning of cycle 7 [min]
a'
Pol-II-S2p
Pol-II-S5p
Normalized Mean$_{nuc}$
Single cell pseudotime from the beginning of cycle 7 [min]
b
Cycle 8
ccp-cens-asympt r²=0.66
ccp-asympt r²=0.66
ccp, ccp-feats r²=0.69
ccp-power r²=0.56
ccp-cens-power r²=0.56
ccp-feats r²=0.66
ccp r²=0.49
ccp-spline r²=0.68
ELPD (log) x10⁴
Cycle 11
ccp-asympt r²=0.76
ccp-cens-asympt r²=0.76
ccp-power r²=0.68
ccp-cens-power r²=0.68
ccp-feats r²=0.66
ccp r²=0.56
ccp, ccp-feats r²=0.71
ccp-spline r²=0.75
ELPD (log) x10⁵
Cycle 12
ccp-asympt r²=0.67
ccp-cens-asympt r²=0.68
ccp-power r²=0.57
ccp- feats r²=0.58
ccp-cens-power r²=0.57
ccp r²=0.46
ccp, ccp-feats r²=0.60
ccp-spline r²=0.64
ELPD (log) x10⁵
c
EVL cells
Mean normalized to cycle 7
Pol-II-S2p
Pol-II-S5p
Cycles
H3K27ac
Pou5f3
Nanog
H2B
DAPI
PCNA
Cycles
d
Drop in r²
Pou5f3
Nanog
PCNA
H3K27ac
H2B
e
Cycle 8
zga, ccp-intensity, morphology, ccp-asympt r²=0.86
zga, morphology, ccp-asympt r²=0.86
zga, ccp-intensity, ccp-asympt r²=0.85
zga, ccp-asympt r²=0.85
ELPD (log)
Cycle 9
zga, ccp-intensity, morphology, ccp-asympt r²=0.88
zga, ccp-intensity, morphology, ccp-asympt r²=0.88
zga, morphology, ccp-asympt r²=0.86
zga, ccp-asympt r²=0.86
ELPD (log)
Cycle 10
zga, ccp-intensity, morphology, ccp-asympt r²=0.69
zga, ccp-intensity, ccp-asympt r²=0.68
zga, ccp-asympt r²=0.62
zga, morphology, ccp-asympt r²=0.63
ELPD (log)
Cycle 11
zga, ccp-intensity, morphology, ccp-asympt r²=0.82
zga, ccp-intensity, ccp-asympt r²=0.82
zga, morphology, ccp-asympt r²=0.81
zga, ccp-asympt r²=0.80
ELPD (log)
Cycle 12
zga, ccp-intensity, morphology, ccp-asympt r²=0.74
zga, ccp-intensity, ccp-asympt r²=0.73
zga, morphology, ccp-asympt r²=0.73
zga, ccp-asympt r²=0.71
ELPD (log)
f
Cycle 8
zga, ccp-intensity r²=0.79
zga r²=0.78
ccp-intensity r²=0.53
zga, morphology, ccp-intensity r²=0.63
ELPD (log)
Cycle 9
zga, ccp-intensity r²=0.87
zga, morphology, ccp-intensity r²=0.88
zga, morphology r²=0.78
zga r²=0.76
ELPD (log)
Cycle 10
zga, ccp-intensity r²=0.64
zga, morphology, ccp-intensity r²=0.66
zga r²=0.59
zga, morphology r²=0.61
ELPD (log)
Cycle 11
zga, morphology r²=0.77
zga, morphology, ccp-intensity r²=0.78
zga r²=0.71
zga, ccp-intensity r²=0.74
ELPD (log)
Cycle 12
zga, morphology r²=0.69
zga, morphology, ccp-intensity r²=0.69
zga, ccp-intensity r²=0.66
zga r²=0.66
ELPD (log)

Supplementary Fig. 6: Modeling transcriptional activity.

a. Mean nuclear intensities of Pou5f3, H3K27ac, H2B and Nanog across division cycles 7 to 12. Sold lines demarcate Gaussian smoothing of points and dashed lines and color changes indicate cycle transitions.

a'. Mean nuclear intensities of Pol-II-S2p (cyan) and Pol-II-S5p (magenta) across division cycles 7 to 12. Sold lines demarcate Gaussian smoothing of points.

b. Model comparisons using CCP or features used for inferring CCP (CCP features, see Materials and Methods) as linear or non-linear predictors of transcription output measured by mean nuclear Pol-II-S2p (natural log-transformed) for cycles 8, 11 and 12. Models are ranked based on their performance on held-out data using expected log pointwise predictive density ELPD (improving from left to right). R^2 demarcates explained variance for each model. Asymptotic (asympt), power and spline non-linear regression models of CCP were tested. Linear models include CCP or features used for CCP inference (ccp-feats). For the asymptotic and power regressions, additional models were tested where data was clipped at 100 gray values, censoring data points with low signal-to-noise ratios (ccp-cens-asympt, ccp-cens-power, Materials and Methods).

c. Mean interphase changes of transcriptional reporters Pol-II-S5p and Pol-II-S2p (left) and ZGA-associated factors H3K27ac, Pou5f3, Nanog, H2B, and PCNA together with DAPI (right) for EVL cells across division cycles 7 to 12. Error bars, std.

d. Permutation analysis showing the reduction in R^2 for ZGA-associated factors, when predicting Pol II-S2p.

e. Top 4 models in predicting mean nuclear Pol-II-S2p (natural log-transformed) using CCP as non-linear predictors and intensity features used for inferring CCP (CCP-intensity), morphology features and ZGA factors (in short zga) as linear regressor in cycles 8 to 12. Error bars, sem.

f. Top 4 models in predicting mean nuclear Pol-II-S2p (natural log-transformed) using CCP-intensity, morphology and ZGA factors as linear regressor in cycles 8 to 12. Error bars, sem. Same data as in Fig. 1.Source data are provided as a Source Data file.

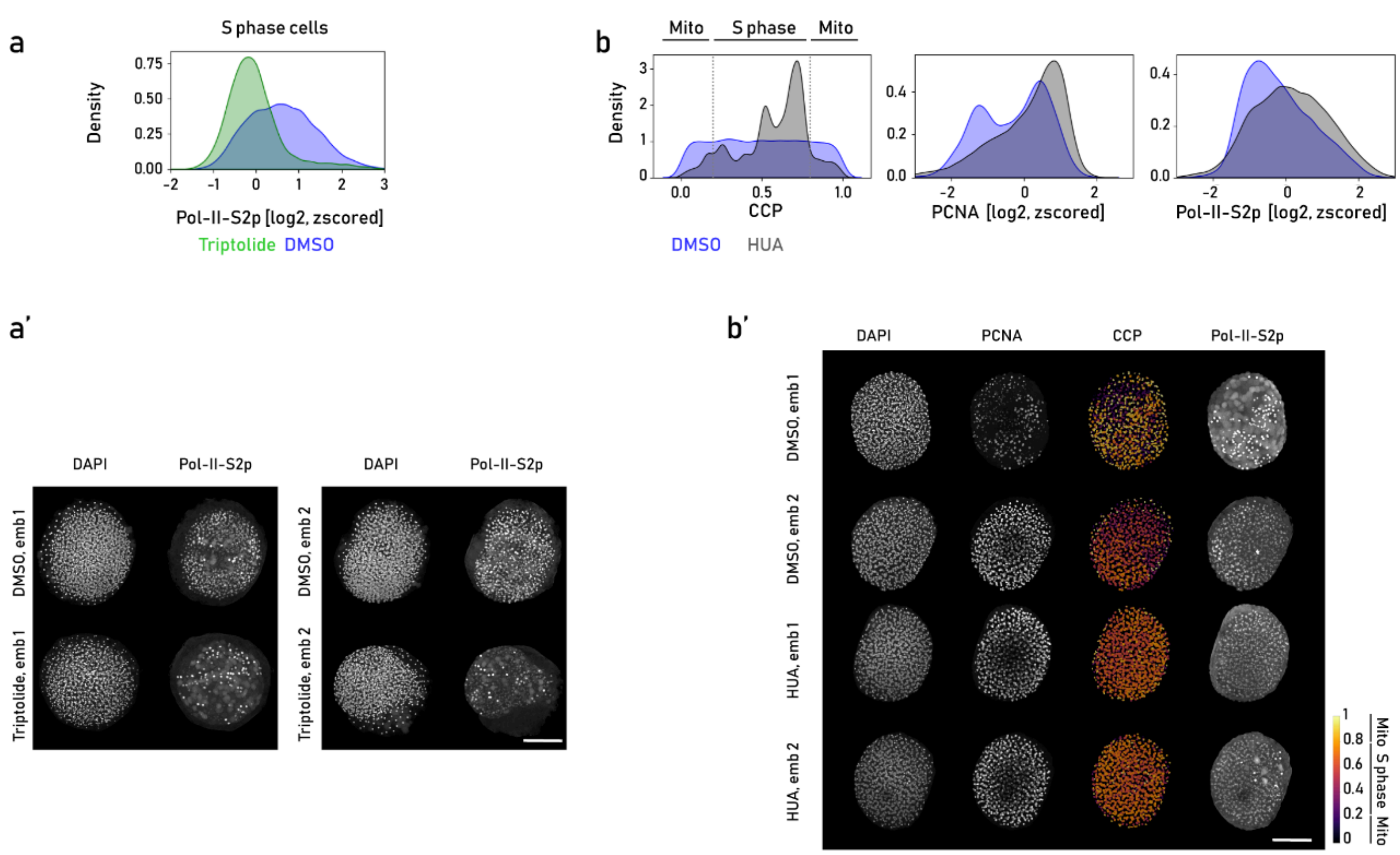

Supplementary Fig. 7: Pharmacological perturbations.

a, Density plot of transcriptional activity, measured by Pol-II-S2p levels, in Triptolide-treated embryos (*N*=1 experiment, *n* = 26 embryos) and DMSO-treated control embryos (*N*=1, *n* = 29).

a′, 3D renderings of DAPI and Pol-II-S2p staining in two representative embryos (emb) from each of the DMSO and Triptolide treatment groups.

b, Density plots of cell cycle phase (left), PCNA levels (middle), and transcriptional activity measured by Pol-II-S2p levels (right) in HUA-treated embryos (*N*=1, *n* = 44) and DMSO-treated control embryos (*N*=1, *n* = 18). Dashed lines in the cell cycle phase plot demarcate the beginning and end of S phase. Mito: Mitosis.

b′, 3D renderings of DAPI, PCNA, inferred CCP, and Pol-II-S2p in two representative embryos from each of the DMSO and HUA treatment groups. Scale bars, 200 μm. Source data are provided as a Source Data file.

Supplementary Tables:

Primary	Host	Dilution	Stain Type	Secondary	Secondary Dilution	Staining Cycle	Catalogue Number	Vendor
Pol-II-S2p	Rat	1:250	Indirect	Donkey anti- Rat 488	1:200	1	04-1571	Sigma-Aldrich
Anti-FLAG	Mouse	1:500	Indirect	Donkey anti- Mouse 568	1:200	1	F1804	Sigma-Aldrich
PCNA	Rabbit	1:250	Indirect	Donkey anti- Rabbit 647	1:200	1	13110	Cell Signaling Technology
H3K27ac	Rabbit	1:500	Direct	Zenon Rabbit 488	1:100	2	ab4729	Abcam
β-Catenin	Mouse	1:250	Indirect	Donkey anti- Mouse 568	1:400	2	2677	Cell Signaling Technology
pH3	Rabbit	1:150	Direct	Zenon Rabbit 647	1:150	2	9701	Cell Signaling Technology
H2B	Rabbit	1:100	Direct	Zenon Rabbit 488	1:250	3	ab134211	Abcam
ALYREF	Mouse	1:250	Direct	Zenon Mouse 555	1:100	3	sc-32311	Santa Cruz Biotechnology
Pol-II-S5p	Rat	1:250	Indirect	Donkey anti- Rat 647	1:200	3	04-1572	Sigma-Aldrich
Nanog	Rabbit	1:280	Direct	Zenon Rabbit 488	1:28	4	GTX132491	GeneTex

Supplementary Table 1: List of primary and their corresponding secondary antibodies used in the multiplexing experiment, their dilutions and staining cycles.

Secondary	Stain Type	Catalogue Number	Vendor
Donkey anti-Rat 488	Indirect	A-21208	Thermo Scientific
Donkey anti-Mouse 568	Indirect	A10037	Thermo Scientific
Donkey anti-Rabbit 647	Indirect	A31573	Thermo Scientific
Donkey anti-Rat 647	Indirect	A48272	Thermo Scientific
Zenon Rabbit Kit 488	Direct	Z25302	Thermo Scientific
Zenon Mouse Kit 555	Direct	Z25051	Thermo Scientific
Zenon Rabbit Kit 647	Direct	Z25308	Thermo Scientific

Supplementary Table 2: List of secondary antibodies and Fab fragment labeling kits used in the multiplexing experiment.

**Component**	**Features**	**Description**
**Regression target**		
	Pol2.S2p	Pol-II-S2p (transcription elongation)
**Linear predictors**		
ccp	CCP	Interphase CCP
ccp-features (ccp feats)	PCNA, DAPI, nuclei volume, nucleus/cytoplasmic volume ratio, nuclei roundness, nuclei flatness	A subset of features used for cell cycle phase inference.
ccp-intensity	PCNA, DAPI	Only intensity features used for cell cycle phase inference.
morphology	nuclei volume, cell volume, nucleus/cytoplasmic volume ratio, nuclei roundness, nuclei flatness	Morphology-related features
zga	Pou5f3, Nanog, H3K27ac, H2B	ZGA-associated factors
**Non-linear predictors**		
ccp-asympt	CCP (a,b,c), equation 4	Asymptotic regression on CCP
ccp-power	CCP (a, b), equation 5	Power curve regression on CCP
ccp-spline	Bs (CCP, 3, fit_intercept=False)	B-spline encoded interphase CCP (3 knots without intercept)

Supplementary Table 3: Feature sets used in regression modeling. Linear predictors included CCP, cell cycle features and ZGA factors. Non-linear CCP predictors included B-spline, asymptotic, and power regressions. Model names indicate included feature sets, e.g., *zga_ccp-asympt* combines four ZGA predictors with asymptotic CCP regression (7 parameters in total).
