# Supplementary material for: Multiplexed embryo profiling links cellular state to zygotic genome activation in single cells
Source: Nat Commun. 2026 Sep 17;17:10929. doi: 10.1038/s41467-026-77784-7 (PMC13680386; doi:10.1038/s41467-026-77784-7)
Supplement: Supplementary file 2 [file 41467_2026_77784_MOESM2_ESM.pdf]

nature portfolio

Corresponding author(s): Shamipour and Pelkmans

Last updated by author(s): May 27, 2026

Reporting Summary

Nature Portfolio wishes to improve the reproducibility of the work that we publish. This form provides structure for consistency and transparency in reporting. For further information on Nature Portfolio policies, see our Editorial Policies and the Editorial Policy Checklist.

Statistics

For all statistical analyses, confirm that the following items are present in the figure legend, table legend, main text, or Methods section.

n/a	Confirmed	
☐	☒	The exact sample size (*n*) for each experimental group/condition, given as a discrete number and unit of measurement
☒	☐	A statement on whether measurements were taken from distinct samples or whether the same sample was measured repeatedly
☒	☐	The statistical test(s) used AND whether they are one- or two-sided<br>*Only common tests should be described solely by name; describe more complex techniques in the Methods section.*
☒	☐	A description of all covariates tested
☐	☒	A description of any assumptions or corrections, such as tests of normality and adjustment for multiple comparisons
☐	☒	A full description of the statistical parameters including central tendency (e.g. means) or other basic estimates (e.g. regression coefficient) AND variation (e.g. standard deviation) or associated estimates of uncertainty (e.g. confidence intervals)
☒	☐	For null hypothesis testing, the test statistic (e.g. *F*, *t*, *r*) with confidence intervals, effect sizes, degrees of freedom and *P* value noted<br>*Give P values as exact values whenever suitable.*
☐	☒	For Bayesian analysis, information on the choice of priors and Markov chain Monte Carlo settings
☒	☐	For hierarchical and complex designs, identification of the appropriate level for tests and full reporting of outcomes
☐	☒	Estimates of effect sizes (e.g. Cohen's *d*, Pearson's *r*), indicating how they were calculated

Our web collection on statistics for biologists contains articles on many of the points above.

Software and code

Policy information about availability of computer code

Data collection	Data were collected by automated spinning-disk confocal imaging of fixed embryos on a Yokogawa CV8000 microscope and by live imaging on an inverted VisiScope spinning-disk microscope. For the multiplexed dataset, embryos were detected and selected using a custom Python 3.9 script before volumetric image acquisition.
Data analysis	Data analysis was performed using custom code in Python 3.9 and established bioimage-analysis and statistical packages. The workflow included elastix/SimpleElastix for image registration, ilastik for pixel classification, Cellpose for nuclear segmentation, scikit-image for cell segmentation, the BaSiC Fiji plugin for flatfield correction, and a custom napari feature-classifier plugin for interactive label annotation and random-forest classification. Dimensionality reduction and preprocessing used scikit-learn-compatible methods including UMAP, PCA, Palantir and standard scaling/transformations; statistical analyses used SciPy, and Bayesian regression modeling used PyMC, Bambi and ArviZ. PyVista was used to generate streamlines for pseudowave visualization. For the live-imaging validation experiment, nuclei were manually tracked using Mastodon in Fiji.

For manuscripts utilizing custom algorithms or software that are central to the research but not yet described in published literature, software must be made available to editors and reviewers. We strongly encourage code deposition in a community repository (e.g. GitHub). See the Nature Portfolio guidelines for submitting code & software for further information.

Data

Policy information about availability of data

All manuscripts must include a data availability statement. This statement should provide the following information, where applicable:

- Accession codes, unique identifiers, or web links for publicly available datasets
- A description of any restrictions on data availability
- For clinical datasets or third party data, please ensure that the statement adheres to our policy

All source data generated in this study is publicly available on BioImage Archive under the Accession number S-BIAD3228.

Research involving human participants, their data, or biological material

Policy information about studies with human participants or human data. See also policy information about sex, gender (identity/presentation), and sexual orientation and race, ethnicity and racism.

Reporting on sex and gender	not applicable.
Reporting on race, ethnicity, or other socially relevant groupings	not applicable.
Population characteristics	not applicable.
Recruitment	not applicable.
Ethics oversight	not applicable.

Note that full information on the approval of the study protocol must also be provided in the manuscript.

Field-specific reporting

Please select the one below that is the best fit for your research. If you are not sure, read the appropriate sections before making your selection.

[x] Life sciences [ ] Behavioural & social sciences [ ] Ecological, evolutionary & environmental sciences

For a reference copy of the document with all sections, see nature.com/documents/nr-reporting-summary-flat.pdf

Life sciences study design

All studies must disclose on these points even when the disclosure is negative.

Sample size	The main multiplexed imaging dataset comprised 212 early zebrafish embryos collected between 2 and 4 hpf at 15-minute intervals, corresponding to division cycles 7-12, and contained 458,826 individual cells in total. Embryos from different time points were pooled and distributed across 30 wells (approximately 7-9 embryos per well) to minimize technical bias. Additional validation and perturbation experiments included 39 tracked nuclei from 2 live-imaged embryos, triptolide-treated embryos (n = 26) and DMSO controls (n = 29), and HUA-treated embryos (n = 44) with DMSO controls (n = 18). The manuscript does not report an a priori power calculation for sample size determination.
Data exclusions	Exclusion criteria were defined during image-analysis and modeling. Cells with nuclear DAPI correlation below 0.9 between acquisition cycles after registration were excluded from downstream analysis. A classifier was used to identify and remove debris, and YSL cells from downstream analyses because of their distinct nuclear morphology. For CCP inference, additional outlier removal was applied to exclude label objects with fluorescence artifacts or mis-segmented nuclei. In predictive analyses and example embryo visualizations, YSL nuclei, mitotic cells, segmentation artifacts, and intensity outliers were excluded where indicated.
Replication	The main dataset spans 212 embryos across developmental time. As a technical control for staining reproducibility, markers from the first three staining cycles were re-stained in cycle four using three replicate wells each, and concordance was assessed at both the mean nuclear intensity and single-pixel levels.
Randomization	To reduce technical variability, embryos from different developmental time points were pooled and randomly distributed within wells before imaging. This random distribution was used to minimize well- or batch-associated technical bias across developmental stages.
Blinding	The manuscript does not report blinding during sample processing, imaging, annotation, or analysis. Samples from different conditions were processed according to the same experimental workflow.

Reporting for specific materials, systems and methods

We require information from authors about some types of materials, experimental systems and methods used in many studies. Here, indicate whether each material, system or method listed is relevant to your study. If you are not sure if a list item applies to your research, read the appropriate section before selecting a response.

Materials & experimental systems

n/a	Involved in the study
☐	☒ Antibodies
☒	☐ Eukaryotic cell lines
☒	☐ Palaeontology and archaeology
☐	☒ Animals and other organisms
☒	☐ Clinical data
☒	☐ Dual use research of concern
☒	☐ Plants

Methods

n/a	Involved in the study
☒	☐ ChIP-seq
☒	☐ Flow cytometry
☒	☐ MRI-based neuroimaging

Antibodies

Antibodies used	Whole-mount multiplexed immunofluorescence was performed using the following primary antibodies: Pol-II-S2p (rat, 1:250, Sigma-Aldrich, 04-1571), anti-FLAG as a readout for Pou5f3 in the pou5f3-3xflag line (mouse, 1:500, Sigma-Aldrich, F1804), PCNA (rabbit, 1:250, Cell Signaling Technology, 13110), H3K27ac (rabbit, 1:500, Abcam, ab4729), β-catenin (mouse, 1:250, Cell Signaling Technology, 2677), pH3 (rabbit, 1:150, Cell Signaling Technology, 9701), H2B (rabbit, 1:100, Abcam, ab134211), ALYREF (mouse, 1:250, Santa Cruz Biotechnology, sc-32311), Pol-II-S5p (rat, 1:250, Sigma-Aldrich, 04-1572), and Nanog (rabbit, 1:280, GeneTex, GTX132491). For high-abundance targets, directly labeled primary antibodies were generated using Zenon labeling kits; other stainings were performed by indirect immunofluorescence.<br>Secondary reagents were donkey anti-rat 488 (Thermo Scientific, A-21208, 1:200), donkey anti-mouse 568 (Thermo Scientific, A10037, 1:200 or 1:400 for β-catenin), donkey anti-rabbit 647 (Thermo Scientific, A31573, 1:200), Zenon Rabbit Kit 488 (Thermo Scientific, Z25302), Zenon Mouse Kit 555 (Thermo Scientific, Z25051), and Zenon Rabbit Kit 647 (Thermo Scientific, Z25308).
Validation	As a technical control for staining reproducibility, markers from the first three staining cycles were re-stained in the final cycle using replicate wells, and correlations between original and repeated stainings were assessed at both mean nuclear intensity and single-pixel levels. These antibodies have been tested in Berry et al, 2022 and Kramer et al, 2022 and doi: 10.1126/sciadv.abg4243)

Animals and other research organisms

Policy information about [studies involving animals](); [ARRIVE guidelines]() recommended for reporting animal research, and [Sex and Gender in Research]()

Laboratory animals	Zebrafish (Danio rerio) embryos were used in this study, including wild-type and a homozygous pou5f3-3xflag transgenic line generated by CRISPR/Cpf1 and maintained by in-crossing identified homozygous fish. Embryos were collected between 2 and 4 hours post-fertilization and raised at 28 °C in E3 medium until the required developmental stage.
Wild animals	The study did not involve wild animals.
Reporting on sex	Sex was not determined / not considered, as the study was performed on early zebrafish embryos collected at 2–4 hpf, before sex differentiation is relevant to the experimental design.
Field-collected samples	The study did not involve field-collected samples.
Ethics oversight	Zebrafish were maintained in the fish facility of Prof. Darren Gilmour. All animal work was carried out according to FELASA guidelines and standards of the University of Zurich. The pou5f3-3xflag transgenic line was generated in the laboratory of Prof. Nadine Vastenhouw at the University of Lausanne according to Swiss regulations (canton Vaud, license number VD-H28).

Note that full information on the approval of the study protocol must also be provided in the manuscript.

Plants

Seed stocks	not applicable.
Novel plant genotypes	not applicable.
Authentication	not applicable.
