# Supplementary material for: Multiplexed embryo profiling links cellular state to zygotic genome activation in single cells
Source: Nat Commun. 2026 Sep 17;17:10929. doi: 10.1038/s41467-026-77784-7 (PMC13680386; doi:10.1038/s41467-026-77784-7)
Supplement: Supplementary file 3 [file 41467_2026_77784_MOESM3_ESM.pdf]

nature portfolio

Peer Review File

Multiplexed embryo profiling links cellular state to zygotic genome activation in single cells

Corresponding Author: Dr Shayan Shami Pour

This file contains all reviewer reports in order by version, followed by all author rebuttals in order by version.

Version 0:

Reviewer comments:

Reviewer #1

(Remarks to the Author)
Hess et al establish a pipeline for large-scale iterative immunostaining of the zebrafish embryo and the compilation and analysis of the resulting large datasets in a streamlined manner. They apply this pipeline to determine several aspects of the cell state, ranging from its cell cycle phase to zygotic genome activation, and reveal the predictive power of cell cycle phase (CCP) and the abundance of DNA replication and pluripotency factors, namely Nanog and Pou5f3, while determining cellular transcriptional activity. The study is well-performed, the results are interpreted accurately, and presented without overstating the findings. It will be an excellent addition to the field, providing a valuable toolkit for not only cell state and ZGA analysis but also for use cases beyond these. However, I have a couple of suggestions to strengthen the study further.
1. While I agree that CCP and other factors (Pou5f3, PCNA, Nanog, etc.) can be used to predict the transcriptional activity of a cell, it would be good to show that there is, in fact, a causal relationship between these factors and transcription. For example, since CCP is the major predictor, it could be perturbed by mosaically slowing down or accelerating cell cycle progression to see if transcriptional activity is altered. In addition, this would be useful to ascertain the influence of CCP on DNA replication and pluripotency factors, which also impact transcription. These analyses would help unequivocally establish if, for instance, CCP and transcriptional activity co-vary under the influence of a third factor or if CCP itself influences transcriptional activity.

2. In lines 252-254, the authors state: “Together, these results demonstrate that transcriptional heterogeneity during ZGA in early zebrafish embryos emerges from the interplay of within-cycle variations shaped by CCP and across-cycle accumulation of pluripotency factors and chromatin activation marks.”

However, as explained in comment 1, while a predictive power is established, which, although it suggests that a causal relationship is likely, is in itself insufficient to claim that transcriptional heterogeneity emerges out of CCP and factor accumulation variations. I strongly suggest that the sentence be revised to reflect this idea or that a causal relationship be demonstrated.

(Remarks on code availability)
-

Reviewer #2

(Remarks to the Author)
In their manuscript, “Multiplexed embryo profiling links cellular state to zygotic genome activation in single cells,” Hess et al. present a method that is inspired by a procedure developed and widely used for two-dimensional samples such as cell cultures and tissue sections. The method is based on sequential antibody staining that reports on the expression pattern of multiple antigens at the whole embryo, tissue, cell, and subcellular levels. The experimental conditions presented by the authors allowed them to derive such information from antibody staining performed on 3D samples of very early embryonic stages in zebrafish. Combined with a strong computer vision-assisted analysis pipeline, the method allowed the authors to identify temporal and spatial relationships among different protein stainings that report on cell cycle progression, pluripotency state, chromatin structure, transcriptional activity etc, and proteins that control these parameters. From my point of view, the results are convincing, and many controls increase the confidence in the accuracy and robustness of the

pipeline established by the authors.

At the biological level, the findings shed light on the mechanisms controlling zygotic gene activation and cell division patterns by simultaneously incorporating a very large number of parameters and defining relationships among them. While some statements could be/have been made by employing standard, currently used techniques, without the method presented here, one has to stitch together data from different low-throughput experiments to reach similar conclusions. The unbiased, holistic approach allowed the authors to identify new relationships among activities, cellular events, and structural features with high confidence. In future work, the mechanistic basis for the relationships identified here and their functional importance will be investigated.

Considering the broad spectrum of Nature Communications readership, the paper's strength lies largely in the method, which has the potential to improve understanding of similar and other events in zebrafish embryos and other models.
I am listing some comments that I hope the authors find useful when revising the paper. Addressing at least some of the points experimentally or in the text would enhance the impact of the paper.

1. The paper's impact would be strengthened by incorporating anti-GFP/anti-protein tag antibodies to demonstrate the method's broader applicability. Additionally, using antibodies against proteins with differentiation signatures distinct from those in the ZGA would show the method's versatility across different biological contexts. Such experiments should Not involve a thorough analysis as performed for the ZGA.
2. Implementing this suggestion would require proof of principle at later developmental stages, which are more widely studied by other laboratories.
3. The paper would benefit from including manipulations such as mutants or overexpression/reducing the activity of proteins regulating relevant processes. This would demonstrate the power of the procedure in defining the basis and nature of phenotypes in an unbiased way.
4. Define CCP the first time it is mentioned, as this abbreviation has different meanings in different contexts.

(Remarks on code availability)

Reviewer #3

(Remarks to the Author)
Hess et al. present a powerful multiplex whole-embryo profiling technology and use it to analyze cellular states during zygotic genome activation. The study is interesting but needs to address the following points.

1. There is much focus on cell cycle phase, but the data is rich in additional parameters that might provide important information and interesting correlations. Please include analysis of nucleo-cytoplasmic ratio and cell size – properties that have been implicated in ZGA.

2. Fig. 1b: please provide stage information.

3. Fig. 1c-e: please provide stage information.

4. Fig. 3d: Why are there no mitotic waves in 7 and 8?

5. Fig. 3g: why are there no mitotic waves in 8 and 9? More generally, the paper would profit from a closer comparison to published in vivo imaging data.

6. Fig. 4a: why are the measurements during mitotic phase so variable?

7. Do you see evidence that S5p (initiation) precedes S2p (elongation)?

8. Fig. 4: h' and f' (anti-correlation of PCNA and pol-II) contradict a (correlation of PCNA and pol-II).

9. The regression models are interesting but how robust are the predictions? What happens if some parameter inputs are permutated.

10. Were there no differences between EVL and deep cells? In the current analyses, no distinction seems apparent, despite their very different morphologies and development. Please clarify.

11. What contributes to transcriptional activity at cycle 7 and why is it so high?

12. The discussion is almost devoid of references to other studies. Please put your results in scholarly context.

Minor comments:

13. Line 58 vs 79: "indirect immunofluorescence" seems to contradict "directly labeling primary antibodies with fluorescently

tagged Fab fragments”.

14. Line 84: “assuming uniform sampling”. In hindsight, is this assumption correct?

15. Abstract, introduction and other places: “causal factors” is an oversell. None of the data presented here establishes causality.

(Remarks on code availability)
Mireia Codina Tobias and Yinan Wan from my lab co-reviewed with me.

Version 1:

Reviewer comments:

Reviewer #1

(Remarks to the Author)
The manuscript has been revised along the lines suggested by the different referees. The revised version has considerably improved over the original version and appears now ready for publication.

(Remarks on code availability)
-

Reviewer #2

(Remarks to the Author)
The authors addressed the points suggested for improving the manuscript and, from my side, the paper is acceptable for publication in the journal.

(Remarks on code availability)

Reviewer #3

(Remarks to the Author)
The authors have addressed my concerns.

(Remarks on code availability)

Open Access This Peer Review File is licensed under a Creative Commons Attribution 4.0 International License, which

permits use, sharing, adaptation, distribution and reproduction in any medium or format, as long as you give appropriate credit to the original author(s) and the source, provide a link to the Creative Commons license, and indicate if changes were made.
In cases where reviewers are anonymous, credit should be given to 'Anonymous Referee' and the source.
The images or other third party material in this Peer Review File are included in the article’s Creative Commons license, unless indicated otherwise in a credit line to the material. If material is not included in the article’s Creative Commons license and your intended use is not permitted by statutory regulation or exceeds the permitted use, you will need to obtain permission directly from the copyright holder.
To view a copy of this license, visit https://creativecommons.org/licenses/by/4.0/

Reviewer #1:

Hess et al establish a pipeline for large-scale iterative immunostaining of the zebrafish embryo and the compilation and analysis of the resulting large datasets in a streamlined manner. They apply this pipeline to determine several aspects of the cell state, ranging from its cell cycle phase to zygotic genome activation, and reveal the predictive power of cell cycle phase (CCP) and the abundance of DNA replication and pluripotency factors, namely Nanog and Pou5f3, while determining cellular transcriptional activity. The study is well-performed, the results are interpreted accurately, and presented without overstating the findings. It will be an excellent addition to the field, providing a valuable toolkit for not only cell state and ZGA analysis but also for use cases beyond these. However, I have a couple of suggestions to strengthen the study further.

1. While I agree that CCP and other factors (Pou5f3, PCNA, Nanog, etc.) can be used to predict the transcriptional activity of a cell, it would be good to show that there is, in fact, a causal relationship between these factors and transcription. For example, since CCP is the major predictor, it could be perturbed by mosaically slowing down or accelerating cell cycle progression to see if transcriptional activity is altered. In addition, this would be useful to ascertain the influence of CCP on DNA replication and pluripotency factors, which also impact transcription. These analyses would help unequivocally establish if, for instance, CCP and transcriptional activity co-vary under the influence of a third factor or if CCP itself influences transcriptional activity.

We thank the reviewer for this thoughtful and constructive suggestion. The choice of cell cycle phase (CCP) and additional factors measured in our multiplexed analysis (Pou5f3, Nanog, etc.) was informed by previous work demonstrating, through perturbation experiments, that these players are required for zygotic genome activation (ZGA)[1–9]. We fully acknowledge that the analyses presented in the original version of the manuscript were correlative in nature and did not, on their own, establish causality. Our aim was therefore to provide a unified quantitative framework that reveals the relative contributions of these factors to predicting heterogeneities in transcriptional activity during ZGA. Accordingly, we have revised the manuscript to clarify that our approach quantifies candidate functional relationships that require experimental perturbation to establish causality.

To address the reviewer's suggestion, we have now incorporated targeted perturbation experiments aimed at probing the relationship between CCP and transcriptional output. Specifically, we constrained cell cycle progression by treating embryos with an S phase-arresting cocktail consisting of hydroxyurea and aphidicolin (HUA)[10,11]. We then performed immunostaining for elongating RNA polymerase II (Pol-II-S2p; marking active transcription) and the S phase marker PCNA, enabling us to assess how shifts in CCP influence transcriptional activity at the single-cell level.

We find that, compared to DMSO-treated controls, HUA-treated embryos show an increased proportion of S-phase cells, as estimated by both our CCP inference pipeline and PCNA levels (revised Fig. S7b-b'). This shift in CCP distribution is accompanied by a corresponding increase in transcriptional output at the population level. These observations are consistent with a close coupling between CCP and transcriptional activity and support the interpretation that modulation of CCP can influence transcriptional heterogeneity during ZGA. While these experiments do not fully establish direct causality, they provide experimental support for the functional relationship inferred from our multiplexed analysis.

We have incorporated these results into the revised manuscript and clarified that our multiplexing platform establishes functional coupling rather than definitive causality. We further emphasize that targeted perturbation experiments are fully compatible with, and complementary to, our multiplexing approach for dissecting the multimodal mechanisms underlying complex biological processes.

2. In lines 252-254, the authors state: "Together, these results demonstrate that transcriptional heterogeneity during ZGA in early zebrafish embryos emerges from the interplay of within-cycle variations shaped by CCP and across-cycle accumulation of pluripotency factors and chromatin activation marks."

However, as explained in comment 1, while a predictive power is established, which, although it suggests that a causal relationship is likely, is in itself insufficient to claim that transcriptional heterogeneity emerges out of CCP and

factor accumulation variations. I strongly suggest that the sentence be revised to reflect this idea or that a causal relationship be demonstrated.

We thank the reviewer for highlighting this point. We agree that the original phrasing overstated the conclusions by implying causality based solely on predictive relationships. While we have now provided additional experimental evidence supporting a role for CCP in shaping transcriptional output (Fig. S7), not all factors examined in our multiplexed analysis have been functionally perturbed in our dataset. We have therefore revised the manuscript to avoid causal language and to frame our findings in terms of functional associations unless directly supported by perturbation experiments.

Reviewer #2:
In their manuscript, "Multiplexed embryo profiling links cellular state to zygotic genome activation in single cells," Hess et al. present a method that is inspired by a procedure developed and widely used for two-dimensional samples such as cell cultures and tissue sections. The method is based on sequential antibody staining that reports on the expression pattern of multiple antigens at the whole embryo, tissue, cell, and subcellular levels. The experimental conditions presented by the authors allowed them to derive such information from antibody staining performed on 3D samples of very early embryonic stages in zebrafish. Combined with a strong computer vision-assisted analysis pipeline, the method allowed the authors to identify temporal and spatial relationships among different protein stainings that report on cell cycle progression, pluripotency state, chromatin structure, transcriptional activity etc, and proteins that control these parameters. From my point of view, the results are convincing, and many controls increase the confidence in the accuracy and robustness of the pipeline established by the authors.
At the biological level, the findings shed light on the mechanisms controlling zygotic gene activation and cell division patterns by simultaneously incorporating a very large number of parameters and defining relationships among them. While some statements could be/have been made by employing standard, currently used techniques, without the method presented here, one has to stitch together data from different low-throughput experiments to reach similar conclusions. The unbiased, holistic approach allowed the authors to identify new relationships among activities, cellular events, and structural features with high confidence. In future work, the mechanistic basis for the relationships identified here and their functional importance will be investigated.
Considering the broad spectrum of Nature Communications readership, the paper's strength lies largely in the method, which has the potential to improve understanding of similar and other events in zebrafish embryos and other models.
I am listing some comments that I hope the authors find useful when revising the paper. Addressing at least some of the points experimentally or in the text would enhance the impact of the paper.
1. The paper's impact would be strengthened by incorporating anti-GFP/anti-protein tag antibodies to demonstrate the method's broader applicability. Additionally, using antibodies against proteins with differentiation signatures distinct from those in the ZGA would show the method's versatility across different biological contexts. Such experiments should Not involve a thorough analysis as performed for the ZGA.
2. Implementing this suggestion would require proof of principle at later developmental stages, which are more widely studied by other laboratories.

We thank the reviewer for the positive and thoughtful evaluation of our work and for recognizing both the technical and biological strengths of the approach. We appreciate the constructive suggestions aimed at further strengthening the manuscript and demonstrating the broader applicability of the method.

Notably, the multiplexing dataset presented in this study was acquired using a Pou5f3-3xFLAG transgenic line together with anti-FLAG immunostaining to quantify Pou5f3 abundance. This already demonstrates the compatibility of our pipeline with antibody detection of tagged proteins. To make this more explicit, we have revised the manuscript to clearly highlight the applicability of our method to anti-protein tag antibodies and similar reporter-based strategies.

In parallel to the present study, we are actively applying 3D-4i to zebrafish embryos at later developmental stages

(4-8 hours post fertilization). In this ongoing work, we multiplex antibodies against downstream effectors of key signaling pathways involved in cell fate specification during gastrulation together with transcription factors marking emerging cell fates, enabling simultaneous capture of multimodal signaling and differentiation states (Reviewer Fig. 1; from Shamipour et al., in preparation). This work aims to elucidate the multimodal signaling combinations that control cell fate decisions *in vivo*.

While we agree that including such proof-of-principle data would further underscore the generalisability of the method, we believe that incorporating these experiments into the current manuscript would substantially expand its scope beyond the central focus on early embryogenesis and ZGA. We therefore prefer to present these results in a dedicated follow-up study, where their biological implications can be explored in appropriate depth. To address the reviewer's suggestion within the present manuscript, we have revised the Discussion to more explicitly highlight the applicability of the method to later developmental stages and broader biological contexts.

A

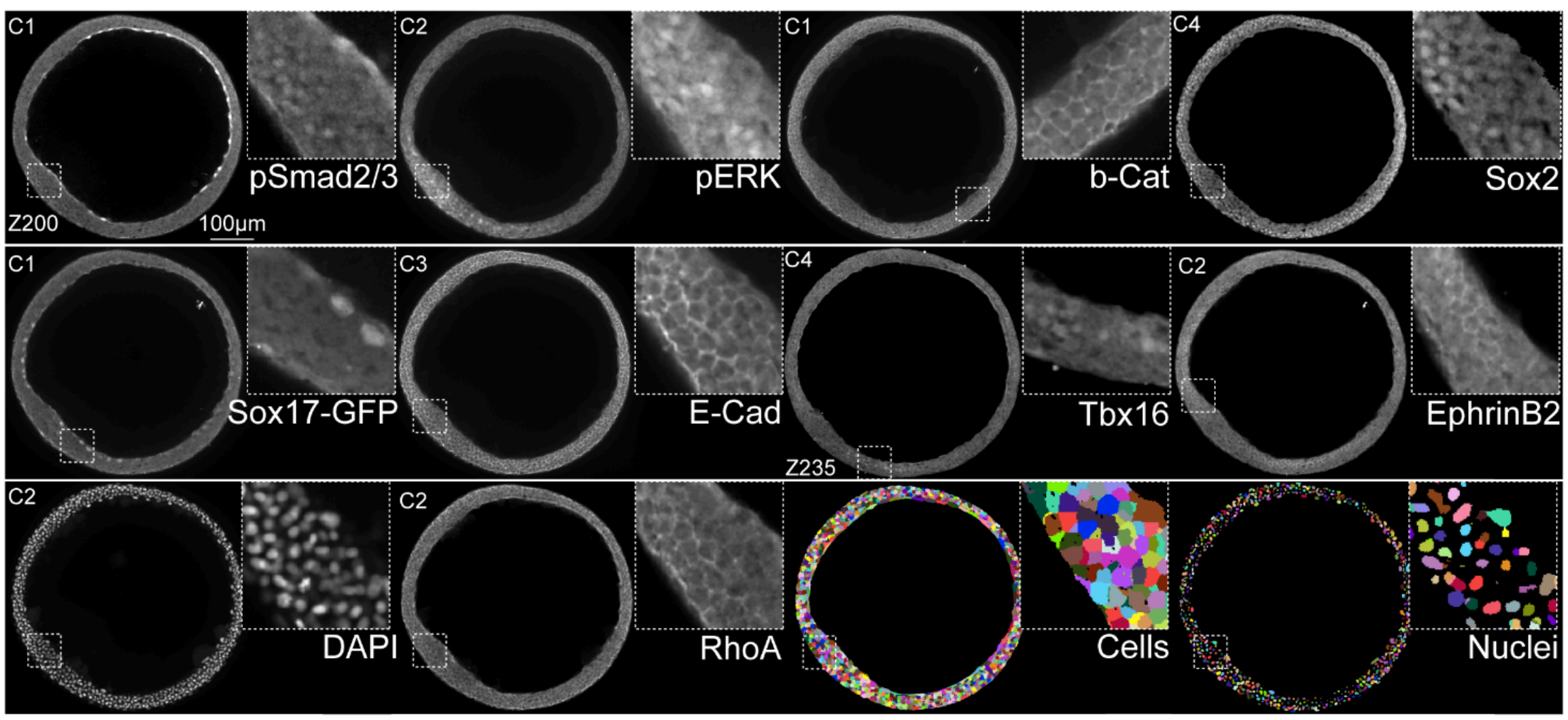

Reviewer Fig. 1:

A. Representative zebrafish embryo at 7 hours post-fertilization stained for signaling pathways, lineage transcription factors, and cellular state markers. Signaling readouts include pSmad2/3 (Nodal/TGFβ), pERK (FGF), and β-catenin (β-cat, Wnt). Lineage markers include Sox2, Sox17 and Tbx16. Additional markers capture adhesion and cytoskeletal components (E-cadherin, RhoA, and EphrinB2). DAPI labels DNA. Cell and nuclear segmentations are shown. Insets display magnified regions. Z200 indicates distance from the animal pole (µm); scale bar, 100 µm. Imaging cycle indices are indicated in the top left of the images.

3. The paper would benefit from including manipulations such as mutants or overexpression/reducing the activity of proteins regulating relevant processes. This would demonstrate the power of the procedure in defining the basis and nature of phenotypes in an unbiased way.

We thank the reviewer for this suggestion. To address this point, we have now incorporated targeted perturbation experiments combining wild-type embryos with embryos subjected to RNA polymerase inhibition (Triptolide)[6] and S phase-arresting cocktail (HUA[11]). Across these conditions, we quantified cell cycle phase, PCNA levels and transcriptional output using our 3D-4i pipeline.

We find that our approach robustly captures transcriptional inhibition following Triptolide treatment (Fig. S7a). In addition, shifting the CCP distribution toward S phase using HUA treatment is associated with an increase in transcriptional activity across cells. Collectively, these results demonstrate the ability of our approach to quantitatively

resolve phenotypic consequences of perturbations in an unbiased, single-cell manner. These data are now included in the revised manuscript (Fig. S7).

4. Define CCP the first time it is mentioned, as this abbreviation has different meanings in different contexts.
We have now defined CCP at its first appearance in the manuscript to avoid ambiguity.

Reviewer #3:
Hess et al. present a powerful multiplex whole-embryo profiling technology and use it to analyze cellular states during zygotic genome activation. The study is interesting but needs to address the following points.

We thank the reviewer for their careful reading of the manuscript and for the constructive comments, which have helped us improve both the scope and clarity of the work. We address each point below.

1. There is much focus on cell cycle phase, but the data is rich in additional parameters that might provide important information and interesting correlations. Please include analysis of nucleo-cytoplasmic ratio and cell size – properties that have been implicated in ZGA.

We thank the reviewer for this suggestion. We have now incorporated nucleo-cytoplasmic ratio, cell size, and nuclear morphology features (collectively referred to as "morphology") into our modeling framework for predicting transcriptional activity. We find that morphology features, together with ZGA factors and CCP intensity-based features, explain a substantial proportion of transcriptional variability within a given division cycle (Fig. S6f). In addition, incorporating morphology features into the previously developed non-linear CCP models results in a modest improvement in prediction accuracy (Fig. S6e). These findings suggest that, although morphology contributes to explaining transcriptional variability, it largely captures information that overlaps with other features already included in the model.

2. Fig. 1b: please provide stage information.
3. Fig. 1c-e: please provide stage information.

We have now specified the developmental stage information for these figures in the main text and/or figure legends.

4. Fig. 3d: Why are there no mitotic waves in 7 and 8?
5. Fig. 3g: why are there no mitotic waves in 8 and 9?
Mitotic waves during earlier division cycles are extremely rapid, resulting in highly synchronized cell cycle phases across the embryo. This is reflected in our circular synchrony measurements (Fig. 3e) and is consistent with live-imaging data reporting very low division cycle duration variance (~0.5-1.5 min^2 for cycles 7-8)[12]. As a result, although mitotic waves are present, their rapid propagation and minimal phase differences make them difficult to resolve in snapshot measurements such as ours, leading to an apparently uniform cell cycle state. To address this more explicitly, we have included additional representative embryos for each division cycle (Fig. 3d' and S4b), where subtle phase differences between neighboring cells can occasionally be observed, consistent with rapidly propagating waves. In addition, the gradient-based approach used to generate streamlines for visualizing mitotic pseudowaves in Fig. 3g is less reliable near tissue boundaries and in early stages with relatively few nuclei[13], as local gradients are then estimated from one-sided and often sparse neighbor configurations. This limitation is now stated in the manuscript.

More generally, the paper would profit from a closer comparison to published in vivo imaging data.
To further validate our CCP inference model and enable a closer comparison with in vivo imaging data, we have now performed live-imaging experiments in embryos injected with histone H1 conjugated to Alexa Fluor 488. We tracked single cells across the 10th division cycle and quantified nucleus size dynamics over time.
Comparing these measurements to our fixed, multiplexed dataset, we observe close agreement between nuclear size dynamics obtained from live imaging and those inferred from our multiplexed data (Fig. S4a-a′), supporting the

accuracy of our approach for inferring cell cycle phase at single-cell resolution from fixed samples. These comparisons are now included in the revised manuscript.

6. Fig. 4a: why are the measurements during mitotic phase so variable?

The apparent variability during mitosis in Fig. 4a arises from plotting low gray values on a logarithmic scale, which inherently spreads small values that would otherwise be compressed near zero on a linear scale. For comparison, we provide the same data replotted on a linear scale in Reviewer Fig. 2. Because the measurements span multiple orders of magnitude, we retained the logarithmic scale in the main figure to more clearly visualize the full dynamic range of the data.

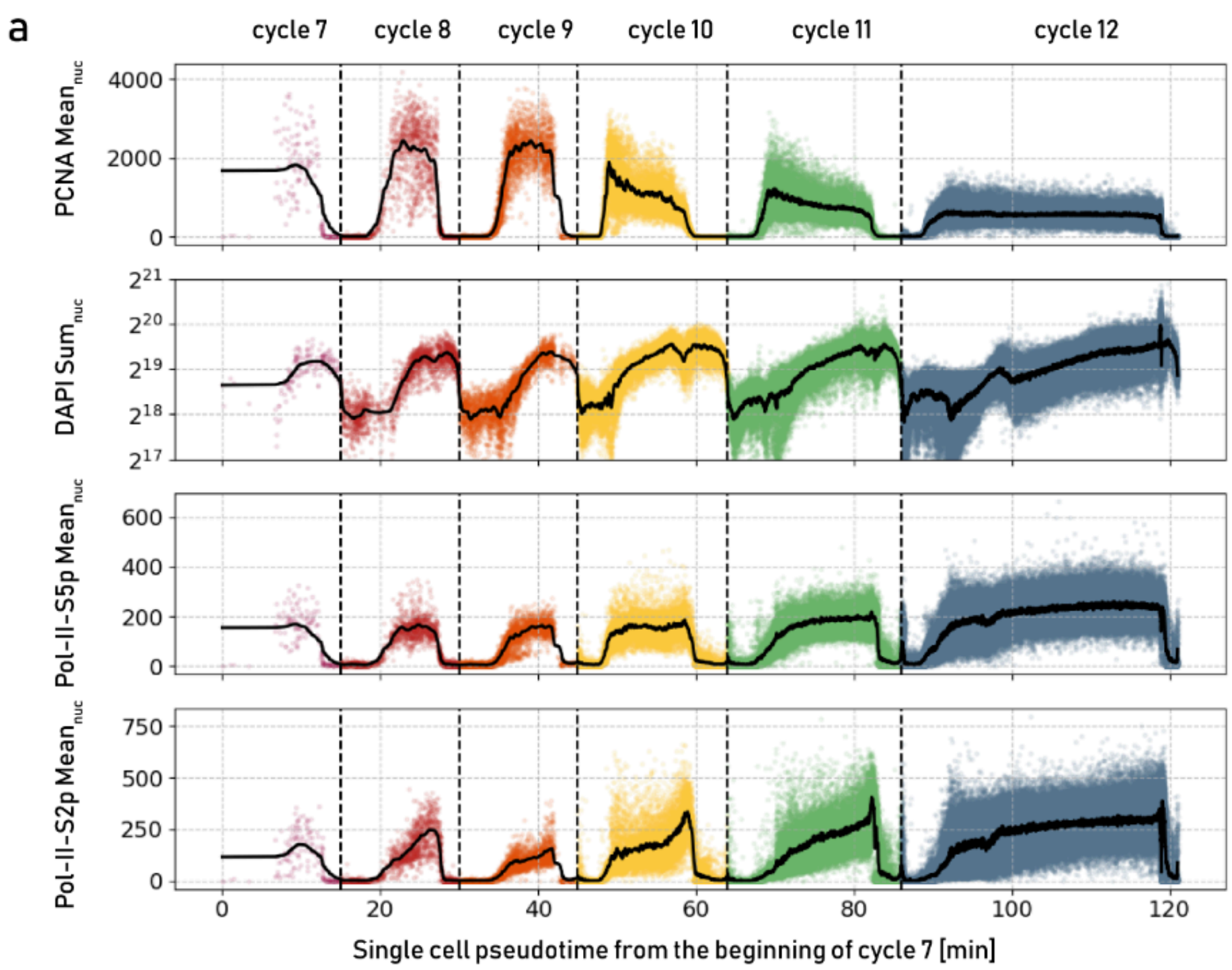

Reviewer Fig. 2: corresponding to main Fig. 4.

a. Mean PCNA, Pol-II-S5p and Pol-II-S2p and sum DAPI nuclear intensities across division cycles 7 to 12 on a linear scale. Sold lines demarcate Gaussian smoothing of points and dashed lines and color changes indicate cycle transitions.

7. Do you see evidence that S5p (initiation) precedes S2p (elongation)?

Yes. We now include a supplementary figure co-plotting Pol-II-S5p and Pol-II-S2p signals (Fig. S6a′), showing that mean Pol-II-S5p levels, marking transcriptional initiation, increase more rapidly and reach a plateau prior to Pol-II-S2p levels, which mark transcriptional elongation, across cell division cycles.

8. Fig. 4: h' and f' (anti-correlation of PCNA and pol-II) contradict a (correlation of PCNA and pol-II).

In Fig. 4f′, the regression coefficient for PCNA is negative, indicating an anti-correlation with Pol-II-S2p. Consistently, we observe opposing trends across division cycles, with transcriptional activity increasing from cycles 9 to 12 while PCNA levels decrease (Fig. 4e). And at the single-cell level, nuclei with higher PCNA levels tend to exhibit lower Pol-II-S2p signals (Fig. 4h'). Altogether, this data is consistent with the anti-correlation between PCNA and Pol-II-S2p.

9. The regression models are interesting but how robust are the predictions? What happens if some parameter inputs are permutated.

To assess the robustness of our regression models, we evaluated their predictive performance using expected log pointwise predictive density (ELPD), which estimates model generalization to unseen data, or 5-fold cross-validation, in which model performance is assessed on 20% held-out data (Materials and Methods). We further confirmed that model performance remains robust when entire embryos are excluded from the training set (Fig. 4g-g′).

In addition, we have now performed permutation analyses in which individual input features are randomly shuffled while predicting transcriptional activity across division cycles. We quantified the resulting drop in explained variance (Fig. S6d). These analyses show that, consistent with its regression coefficient (Fig. 4f'), Pou5f3 makes the strongest contribution to explaining transcriptional variability across cycles among the features tested. These results are now included in the revised manuscript.

10. Were there no differences between EVL and deep cells? In the current analyses, no distinction seems apparent, despite their very different morphologies and development. Please clarify.

For simplicity, our initial analyses inferring cell cycle phase and predicting transcriptional activities combined EVL and deep cells. We have now explicitly compared these populations with respect to transcriptional variability and ZGA-associated factors (Fig. 4e for deep cells and S6c for EVL cells). We find that although they express comparable levels of the studied markers, EVL cells exhibit higher transcriptional activity and active marks of H3K27ac and Pou5f3 level, in line with their early differentiation and epidermal function in the embryo[14]. We have now clarified these points in the manuscript.

11. What contributes to transcriptional activity at cycle 7 and why is it so high?

We observe that mean nuclear Pol-II-S2p levels are already detectable in cycle 7 embryos, whereas discrete active transcription foci are still weak, infrequent, or not consistently resolved. Mean Pol-II-S2p levels then rise progressively, while prominent transcription foci become much more evident by later pre-MBT/MBT stages (Fig. S5). Together with prior work showing that H3K27ac and chromatin accessibility can precede robust Pol-II-S2p accumulation[6,15], this suggests that global transcriptional competence increases gradually, whereas local polymerase concentration at specific loci emerges more sharply as chromatin is remodeled and transcription factor clusters form[16,17].

12. The discussion is almost devoid of references to other studies. Please put your results in scholarly context.

We have substantially expanded the Discussion to include references to prior work and to place our findings in a broader scientific context.

Minor comments:

13. Line 58 vs 79: “indirect immunofluorescence” seems to contradict “directly labeling primary antibodies with fluorescently tagged Fab fragments”.

We have now removed the “indirect” term from our acronym and text, as in our workflow a combination of indirect immunofluorescence and direct labeling of primary antibodies with fluorescently tagged Fab fragments are used.

14. Line 84: “assuming uniform sampling”. In hindsight, is this assumption correct?

Upon further analysis, we find that certain time windows, particularly division cycle 9, are sampled more sparsely. We have revised the text accordingly and discussed this limitation in the revised manuscript.

15. Abstract, introduction and other places: “causal factors” is an oversell. None of the data presented here establishes causality.

We agree with the reviewer. While our analyses quantify the contribution of multiple factors to transcriptional output, they are correlative and predictive in nature. We have revised the manuscript throughout to remove causal language and now explicitly frame our findings as identifying functional associations within a unified quantitative framework. In addition, we have performed targeted perturbation experiments to further probe the relationship between cell cycle progression and transcriptional output (see response to Reviewers 1), while maintaining a cautious interpretation of these results.

References:

1. Heyn, P. *et al.* The Earliest Transcribed Zygotic Genes Are Short, Newly Evolved, and Different across Species. *Cell Reports* **6**, 285–292 (2014).
2. Hadzhiev, Y. *et al.* A cell cycle-coordinated Polymerase II transcription compartment encompasses gene expression before global genome activation. *Nat Commun* **10**, 691 (2019).
3. Kimelman, D., Kirschner, M. & Scherson, T. The events of the midblastula transition in Xenopus are regulated by changes in the cell cycle. *Cell* **48**, 399–407 (1987).
4. Collart, C., Allen, G. E., Bradshaw, C. R., Smith, J. C. & Zegerman, P. Titration of Four Replication Factors Is Essential for the *Xenopus laevis* Midblastula Transition. *Science* **341**, 893–896 (2013).
5. Joseph, S. R. *et al.* Competition between histone and transcription factor binding regulates the onset of transcription in zebrafish embryos. *eLife* **6**, e23326 (2017).
6. Chan, S. H. *et al.* Brd4 and P300 Confer Transcriptional Competency during Zygotic Genome Activation. *Developmental Cell* **49**, 867-881.e8 (2019).
7. Lee, M. T. *et al.* Nanog, Pou5f1 and SoxB1 activate zygotic gene expression during the maternal-to-zygotic transition. *Nature* **503**, 360–364 (2013).
8. Pálfy, M., Schulze, G., Valen, E. & Vastenhouw, N. L. Chromatin accessibility established by Pou5f3, Sox19b and Nanog primes genes for activity during zebrafish genome activation. *PLoS Genet* **16**, e1008546 (2020).
9. Wu, E. & Vastenhouw, N. L. From mother to embryo: A molecular perspective on zygotic genome activation. in *Current Topics in Developmental Biology* vol. 140 209–254 (Elsevier, 2020).
10. Petridou, N. I., Grigolon, S., Salbreux, G., Hannezo, E. & Heisenberg, C.-P. Fluidization-mediated tissue spreading by mitotic cell rounding and non-canonical Wnt signalling. *Nat Cell Biol* **21**, 169–178 (2019).

11. Morita, H. *et al.* The Physical Basis of Coordinated Tissue Spreading in Zebrafish Gastrulation. *Developmental Cell* **40**, 354-366.e4 (2017).

12. Mishra, N., Li, Y. I., Hannezo, E. & Heisenberg, C.-P. Geometry-driven asymmetric cell divisions pattern cell cycles and zygotic genome activation in the zebrafish embryo. *Nat. Phys.* **22**, 139–150 (2026).

13. Meyer, T. H., Eriksson, M. & Maggio, R. C. Gradient Estimation from Irregularly Spaced Data Sets. *Mathematical Geology* **33**, 693–717 (2001).

14. Liu, J. *et al.* Dissecting the regulatory logic of specification and differentiation during vertebrate embryogenesis. Preprint at https://doi.org/10.1101/2024.08.27.609971 (2024).

15. Sato, Y. *et al.* Histone H3K27 acetylation precedes active transcription during zebrafish zygotic genome activation as revealed by live-cell analysis. *Development* **146**, dev179127 (2019).

16. Kuznetsova, K. *et al.* Nanog organizes transcription bodies. *Current Biology* **33**, 164-173.e5 (2023).

17. Miao, L. *et al.* The landscape of pioneer factor activity reveals the mechanisms of chromatin reprogramming and genome activation. *Molecular Cell* **82**, 986-1002.e9 (2022).
